# Antitarget, Anti-SARS-CoV-2 Leads, Drugs, and the Drug Discovery–Genetics Alliance Perspective

**DOI:** 10.1021/acs.jmedchem.2c01229

**Published:** 2023-03-01

**Authors:** Cecilia Pozzi, Anne Vanet, Valeria Francesconi, Lorenzo Tagliazucchi, Giusy Tassone, Alberto Venturelli, Francesca Spyrakis, Marco Mazzorana, Maria P. Costi, Michele Tonelli

**Affiliations:** † Department of Biotechnology, Chemistry and Pharmacy, University of Siena, via Aldo Moro 2, 53100 Siena, Italy; ‡ Université Paris Cité, CNRS, Institut Jacques Monod, F-75013 Paris, France; § Department of Pharmacy, University of Genoa, viale Benedetto XV n.3, 16132 Genoa, Italy; ∥ Department of Life Science, University of Modena and Reggio Emilia, via Campi 103, 41125 Modena, Italy; ⊥ Doctorate School in Clinical and Experimental Medicine (CEM), University of Modena and Reggio Emilia, Via Campi 287, 41125 Modena, Italy; # Department of Drug Science and Technology, University of Turin, Via Giuria 9, 10125 Turin, Italy; ∇ Diamond Light Source, Harwell Science and Innovation Campus, Didcot, Oxfordshire OX11 0DE, U.K.

## Abstract

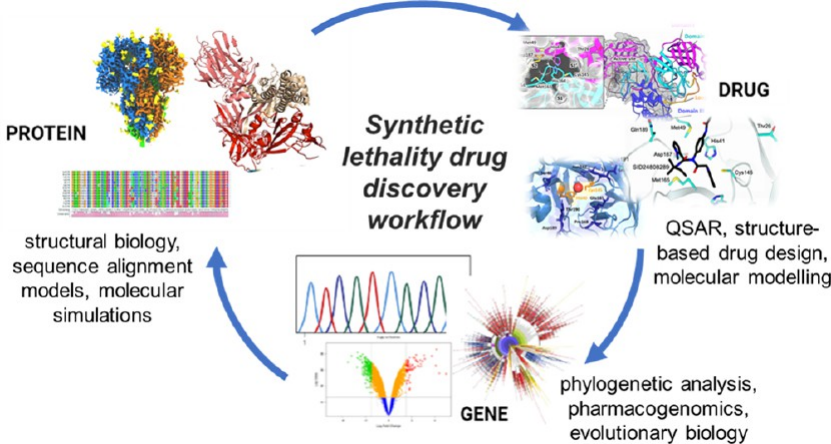

The most advanced antiviral molecules addressing major SARS-CoV-2 targets (Main protease, Spike protein, and RNA polymerase), compared with proteins of other human pathogenic coronaviruses, may have a short-lasting clinical efficacy. Accumulating knowledge on the mechanisms underlying the target structural basis, its mutational progression, and the related biological significance to virus replication allows envisaging the development of better-targeted therapies in the context of COVID-19 epidemic and future coronavirus outbreaks. The identification of evolutionary patterns based solely on sequence information analysis for those targets can provide meaningful insights into the molecular basis of host–pathogen interactions and adaptation, leading to drug resistance phenomena. Herein, we will explore how the study of observed and predicted mutations may offer valuable suggestions for the application of the so-called “synthetic lethal” strategy to SARS-CoV-2 Main protease and Spike protein. The synergy between genetics evidence and drug discovery may prioritize the development of novel long-lasting antiviral agents.

## Introduction

The evolution of SARS-CoV-2, the etiological agent of COVID-19 disease, is characterized by the emergence of mutations that reprogram its transmissibility and pathogenicity, making the antiviral molecules thus far identified vulnerable to the strong drug resistance viral response. To this purpose this review introduces an innovative strategy based on the synergistic cross-talk between drug discovery and genetics for interpreting and preventing drug resistance, thereby increasing drug development success rates. Classical approaches to antiviral therapy reveal some major weaknesses when the mutational characters of the viral or host targets are considered. Known since the beginning of antiviral therapy, drug resistance development should thus be avoided, and new drugs should be conceived to smartly prevent and overcome the unavoidable mutational events. The present review, by taking as an extraordinary timely example the SARS-CoV-2 infection, introduces the novel concept of synthetic lethality (SL) applied to a single target protein (or a group of them) that should be integrated in the discovery paths.

SARS-CoV-2 is an enveloped, positive-sense, single-stranded RNA β-coronavirus of the family Coronaviridae.^1^ Coronaviruses that infect humans historically include several common cold viruses, such as HCoV-OC43, HKU, and 229E. However, over the past two decades, highly pathogenic human coronaviruses have emerged, namely SARS-CoV-1 in 2002, which is associated with 8000 cases worldwide and a death rate of around 10%, and Middle East respiratory syndrome coronavirus (MERS-CoV) in 2012, which caused 2500 confirmed cases and had a death rate of 36%. Infection with these highly pathogenic coronaviruses can result in acute respiratory distress syndrome, which may lead to a long-term reduction of lung function, arrhythmia, or death. In comparison with MERS-CoV or SARS-CoV-1, SARS-CoV-2 has a lower fatality rate but spreads more efficiently, making it difficult to contain. With ∼660 million cases worldwide and approximately 6.9 million deaths by January 12, 2023 (WHO Coronavirus (COVID-19) Dashboard With Vaccination Data) and a death rate in the early pandemic phase (i.e., prevaccine) peaking to 5–10% in many countries, it is one of the largest, unexpected infections in this century and the most concerning one since it found us unprepared. To devise therapeutic strategies counteracting SARS-CoV-2 infection and the associated SARS-CoV-2 pathology, different strategies were proposed since the beginning of the infection spreading, which posed some questions about the efficacy of the drugs used. The countermeasures against this novel coronavirus infection relied on existing antivirals and on repurposing drugs and then on anti-inflammatory and antithrombotic therapies, in line with the growing knowledge on the novel virus.^2^ After two years of cohabitation with SARS-CoV-2, many studies have been conducted that focus on how this coronavirus hijacks the host during infection, to inform the drug discovery and development process. A deep understanding of the structure–function and inhibition of the viral biomolecules and host proteins, triggered after the infection, can suggest new targets and pathways to follow. Many efforts are underway to fight the coronavirus pandemic. The general feeling is that huge international projects will gather many research groups around the world, increasing the chance of identifying a cure.^3^ In the years to follow, probably because of the endemic nature of these viruses’ spread, research efforts will be limited in time; thus the pressure of novel findings and first-in-class cures in the drug discovery field will be uneven. The main projects are related to the essential aspects of the drug discovery process: genetic comparisons; structural biology studies; HTS technologies for rapid antiviral screening; computational biology; repurposing or new chemical entities or halfway discovery (see the FDA on repurposing approaches). The state-of-the-art approach in the case of SARS-CoV-2 infection drug discovery is not satisfactory because mutational events develop drug resistance or intrinsic unresponsiveness by patients, ultimately leading to therapeutic failure.^1^ The identification of evolutionary patterns based on the analysis of sequence information alone for those targets can provide meaningful insights into the molecular basis of host–pathogen interactions and adaptation. The discovery of potential routes of mutations that could lead to new SARS-CoV-2 variants adapting to human hosts and to the new drugs will improve the understanding and monitoring of events critical to tackling pathogens posing worldwide high concern to the public health. Therefore, while thinking of new drugs, the early experimental design should aim to anticipate future resistance response in a concerted effort combining targets’ mutational propensities and the establishment of resilient drug:target interactions.

## Targets and Drugs

The following sections focus on the best-known SARS-CoV-2 targets, namely the main protease (M^pro^, also named 3CL^pro^ and nsp5), the Spike (S) protein, and the RNA-dependent RNA polymerase (RdRp), including the state of the art of the most promising antiviral molecules thus far identified. A comparative analysis of SARS-CoV-2 enzymes/proteins with respect to other human pathogenic CoV homologues and a detailed description of the main chemical features responsible for an efficient target inhibition are proposed here, with the intent to bridge the gap between earlier and current research findings and draw the line of drug discovery strategies in the fight against coronavirus infections.

Major progress was achieved by three COVID-19 antivirals, capable of targeting the M^pro^ (nirmatrelvir–ritonavir) or the RdRp (molnupiravir), which obtained an emergency use authorization or reached the last stage of clinical trials (PF-00835231), timely trying to transform the pandemic context. The evolution of SARS-CoV-2 is characterized by the emergence of sets of mutations occurring in the viral genome that impact virus transmissibility and antigenicity. Therefore, we provide an overview of mutations of the M^pro^, RdRp, and S proteins at the molecular level, in an attempt to help understand how these variants may affect the structural and functional behaviors of SARS-CoV-2 proteins and how they may hamper drug effectiveness.

In this review we explore how the study of the observed and predicted mutations may provide valuable suggestions for the application of the so-called “synthetic lethal” (SL) strategy. This approach aims to develop innovative antiviral drugs able to cause a double mutation by targeting pairs of genes (or pairs of residues) leading to the inactivation of the affected protein and ultimately to virus replicative failure. Within this landscape we have also harmonized the drug target interaction and drug efficacy with the concept of “genetic synthetic lethality” with the intent to offer an instrumental perspective for future coronavirus outbreaks. Although the concept was largely used in anticancer therapy, and more recently in anti-HIV and anti-influenza virus applications, we have dedicated a great focus to the suitability of SARS-CoV-2 M^pro^ and S proteins, whose studies have, to the best of our knowledge, more chances of succeeding.

## Main Protease

Until the first SARS outbreak, the 3C-like protease (3CL^pro^) has emerged as the most druggable target. 3CL^pro^ is more commonly known as main protease (M^pro^) because of its dominant role in the post-translational processing of the ORF1ab polyprotein.

In general, targeting proteases has proven successful in several antiretroviral design campaigns.^4^ In particular, M^pro^ offers several advantages as a drug target:

(i) a highly specific cleavage site (Leu-Gln↓Ser-Ala-Gly), which has never been reported in human hosts, minimizing the risk of off-target effects
(ii) an essential role in the viral replication cycle:^5^ M^pro^ cleaves most structural and nonstructural viral proteins, hence its inhibition would greatly hamper the production of virions, eventually leading to the relief of COVID-19 symptoms
(iii) high structural similarity to SARS-CoV-1 and MERS M^pro^s, which possibly opens the door to the design of pan-coronavirus drugs, also in the case of future outbreaks
(iv) a well-characterized catalytic cycle and large availability of crystallographic data, considerably enriching the possibility of success in either ligand- or structure-based drug design campaigns; indeed, more than 200 crystal structures are currently available in the Protein Data Bank.

**Figure 1:**
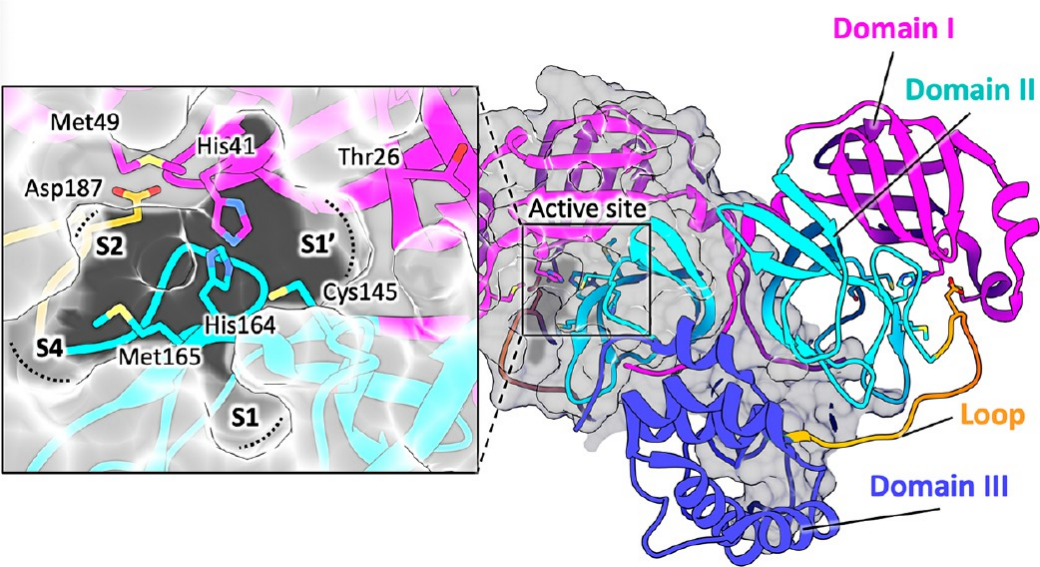
Structure of SARS-CoV-2 M^pro^ (PDB ID 7BUY(12)). The dimeric protein is shown in a cartoon, and one protomer is shown as the gray surface. The three domains are shown in three different colors (domain I in magenta, domain II in cyan, and domain III in blue); the loop connecting domains II and III is in orange. The catalytic site is shown in the inset in the rectangle. Residues are shown as sticks, and subsites S1, S2, S4, and S1′ are indicated over the surface representation of the enzyme.

M^pro^ works as a homodimer, composed of two molecules designated as protomers A and B, each formed by 306 amino acids belonging to three domains (Figure 1).^6,7^ Domains I (residues 8–101) and II (residues 102–184) have an antiparallel β-barrel structure, similar to other CoV proteases and reminiscent of trypsin-like serine-proteases (Figure 1).^6,7^ Domain III (residues 201–303) includes five α-helices arranged into a largely antiparallel globular cluster, connected to domain II by a long loop region (residues 185–200) (Figure 1). The substrate-binding site of SARS-CoV-2 M^pro^ lies in a cleft between domains I and II and features the catalytic dyad Cys145 and His41 (Figure 1). During the catalysis, His41 acts as proton acceptor and Cys145, once deprotonated, is activated for the nucleophilic attack on the carbonyl carbon of the substrate.^8,9^ Thus, it is widely accepted that increased inhibitor potency can be achieved by molecules covalently linking Cys145 and mimicking the intermediate during substrate cleavage.^8^ The substrate-binding pocket is divided into the four main subsites S1′, S1, S2, and S4 (Figure 1), each accommodating the side chain of a single consecutive amino acid of the substrate (generically peptidic (P) fragments P1′ and P1–P3).^6−8^ The S1′ subsite contains the catalytic dyad Cys145 and His41^10,11^ and is also lined by Thr25, Met49, and the backbones of Thr26, Val42, and Thr45 (Figure 1). The subsite S1 of CoV M^pro^, generated by His163, Phe140, and the main chain atoms of Met165, Glu166, and His172, confers absolute specificity for the Gln-P1 residue of the substrate, via two H-bonds. The backbone amides of Gly143 and Cys145 participate in the oxyanion hole, stabilizing the tetrahedral intermediate formed during the cleavage. The deep hydrophobic subsite S2, lined by His41, Met49, Tyr54, Met165, and Asp187 (alkyl part of its side chain), accommodates the hydrophobic residue P2 of the substrate, typically a leucine or a phenylalanine. The substrate residue P3 is usually solvent exposed, preventing the definition of a specific subsite for it. On the other hand, the S4 subsite, accommodating the P4 moiety of the substrate, is defined by the side chains of Met165, Leu166, Phe185, and Gln191.

Domain III of M^pro^ is responsible for the dimeric assembly, as the interface contacting area between the two protomers is mainly localized in this domain. Nonetheless, the N-terminal residues 1–7, known as the N-finger, play an important role in the dimerization and in the formation of the active site of M^pro^.^6,7^ Structural evidence on CoV M^pro^s showed that Ser1 is H-bonded to Glu166 carboxylate and Phe140 backbone carbonyl, both belonging to the S1 subsite of the other protomer.

The expanding knowledge on virus–host interaction opens the way to host-targeting antivirals that should possess a markedly higher barrier for selecting drug-resistant viruses and may provide broad-spectrum antiviral activity when dealing with a cellular target that is recruited by diverse viruses. The interaction of SARS-CoV-2 with host cell proteins is necessary for its successful replication, and cleavage of cellular targets by the main viral protease also may contribute to the disease pathogenesis. The interaction map between SARS-CoV-2 and human proteins has recently been obtained,^13^ thus allowing the identification of some human substrates that are processed by M^pro^, such as histone deacetylase 2 (HDAC2) that mediates the inflammation and interferon response, and tRNA methyltransferase 1 (TRMT1) that catalyzes tRNA modifications for appropriate cellular redox equilibrium. Reduced levels of V-ATPase G1^14^ and NF-kB^15^ proteins were previously reported as a consequence of the proteolytic processing by SARS-CoV-1 M^pro^. *In vitro* proteomic analyses have identified numerous host target proteins, including those involved in the host innate immune response, and proposed cleavage site preferences (for P1-Gln, P2-Leu, and P1′-Gly/Ala/Ser residues) for M^pro^s from SARS-CoV-1, SARS-CoV-2, and HCoV-NL63.^16^ These results further legitimate interest in more in-depth studies to derive a better insight of the molecular mechanisms behind the viral replication, such as the interaction of M^pro^ with the host proteome to evade the innate immune response.

Recent studies have revealed that SARS-CoV-2 M^pro^ acquired 22 mutations in its human host; in the SARS-CoV-2 variants of concern (VOCs), such as Alpha (α, B.1.1.7), Beta (β, B.1.351), Gamma (γ, B1.1.1.28 or P.1), Lambda (λ, B.1.1.1.37/C37), and Omicron (ο, B.1.1.529), the K90R (α, β, γ), G15S (λ), and P132H (ο) M^pro^ mutations are the most recurrent. These mutations are far from key residues responsible for SARS-CoV-2 M^pro^ catalytic activity, substrate binding, and dimerization, so they do not influence the protein functionality.^17^ This suggests that M^pro^ inhibitors may still be exploited as therapeutics also against circulating SARS-CoV-2 variants, as reported for the drug nirmatrelvir in the following section.^18^ However, this scenario depicts the need for further studies to establish the real impact of prevalent variants on M^pro^ cleavage activity and the expected drug efficacy. We advocate here for a stronger alliance between experts in the fields of drug discovery and genetics to achieve better M^pro^ inhibitors.

### Peptidomimetic Inhibitors of M^pro^ Catalytic Site

**Figure 2:**
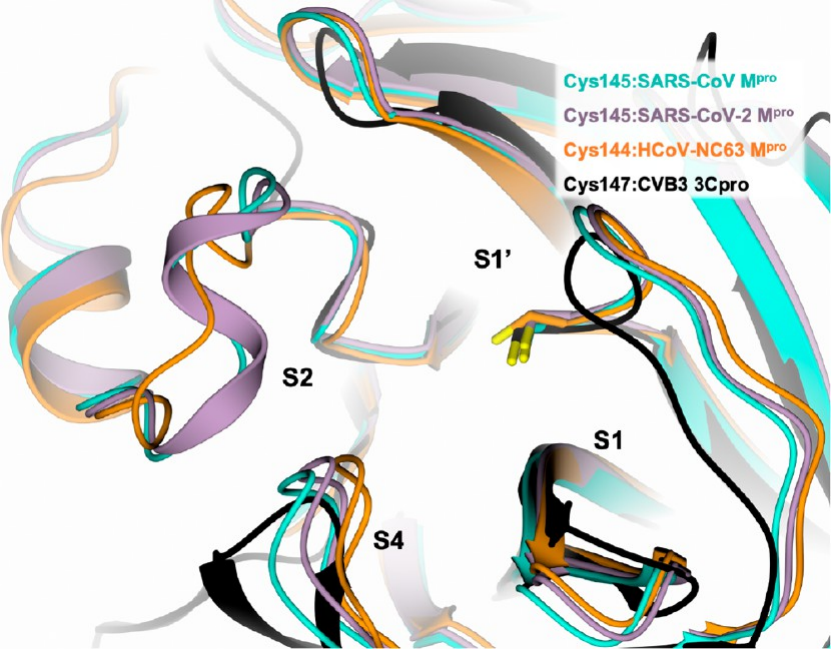
Active site view of the structural comparison among SARS-CoV-1 M^pro^ (cyan, PDB ID 2AMQ(8)), SARS-CoV-2 M^pro^ (lilac, PDB ID 6LU7(19)), HCoV-NL63 M^pro^ (orange, PDB ID 7E6M(20)), and CVB3 3C^pro^ (black, PDB ID 2ZU3(21)). The protein is shown in a cartoon, and the catalytic cysteine is shown in capped sticks.

Recent studies of new CoVs and the accumulation of structural data on CoV M^pro^s from various viruses have shown that the most variable regions are the helical domain III and the surface loops (Figure 2). On the other hand, the substrate-binding pockets are highly conserved among CoV M^pro^’s, suggesting that antiviral inhibitors targeting these sites could have wide spectrum anti-CoV activity.

**Figure 3:**
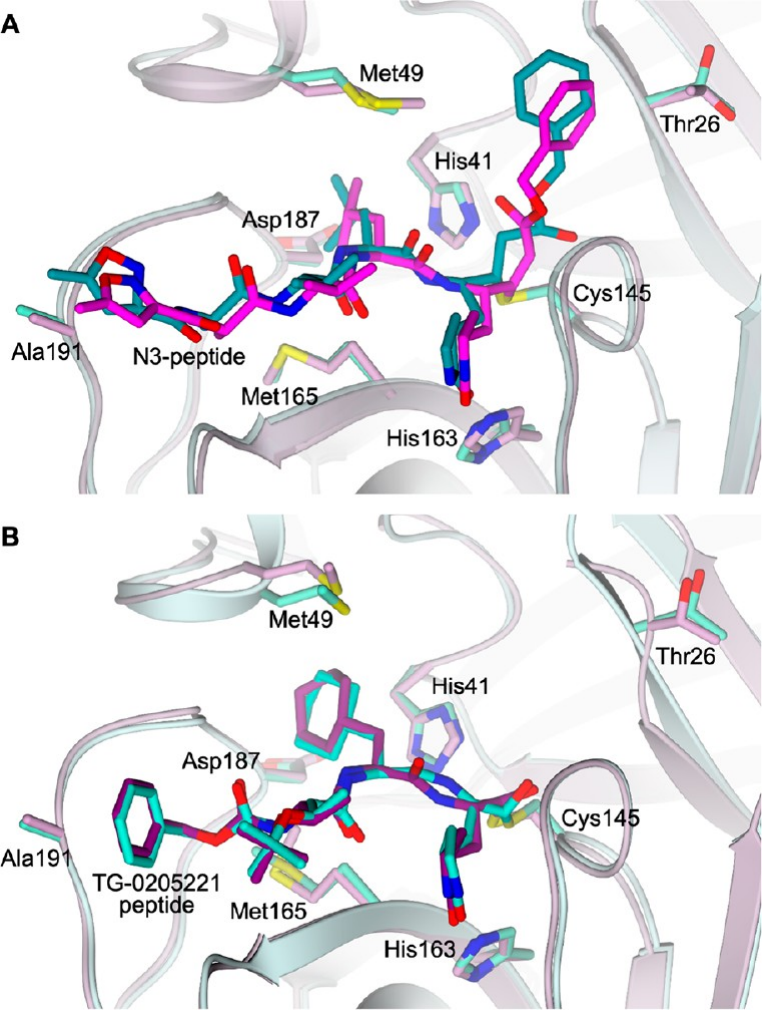
Active site view of the structural comparison between (A) N3 peptide in complex with SARS-CoV-1 M^pro^ (cyan, PDB ID 2AMQ(8)) and SARS-CoV-2 M^pro^ (lilac, PDB ID 6LU7(19)) and (B) TG-0205221 peptide in complex with SARS-CoV-1 M^pro^ (cyan, PDB ID 2GX4(27)) and SARS-CoV-2 M^pro^ (lilac, PDB ID 7C8T(28)). The protein is shown in a cartoon; the ligand and the residues lining the pocket are shown in capped sticks. Oxygen atoms are colored red, nitrogen atoms are blue, and sulfur atoms are yellow.

Indeed, various inhibitors of SARS-CoV-1 and MERS-CoV M^pro^s are also active against SARS-CoV-2 M^pro^.^6,19,22^ This includes for example the Michael acceptor inhibitor N3 (Table 1).^8,19,23−25^ The comparison between the structures of the SARS-CoV-1 and SARS-CoV-2 M^pro^’s in complex with N3 (PDB IDs 2AMQ(8) and 6LU7,^19^ respectively) shows a conserved binding mode within the cavity of both enzymes, supporting a possible pan-CoV activity for this compound^8,25^ (Figure 3A). After forming a covalent bond with the catalytic Cys145 via Michael addition, N3 adopts an extended conformation within the M^pro^ substrate-binding site covering all the key subsites S1, S2, S4, and S1′.^8,19^ Analogous behavior is shown by the peptide TG-0205221 in both complexes (Figure 3B, Table 1). Apart from covalent inhibitors, SARS-CoV-2 M^pro^ has been cocrystallized with noncovalent inhibitors and with a number of fragments bound to the orthosteric and alternative sites.^26^

**Table 1:**
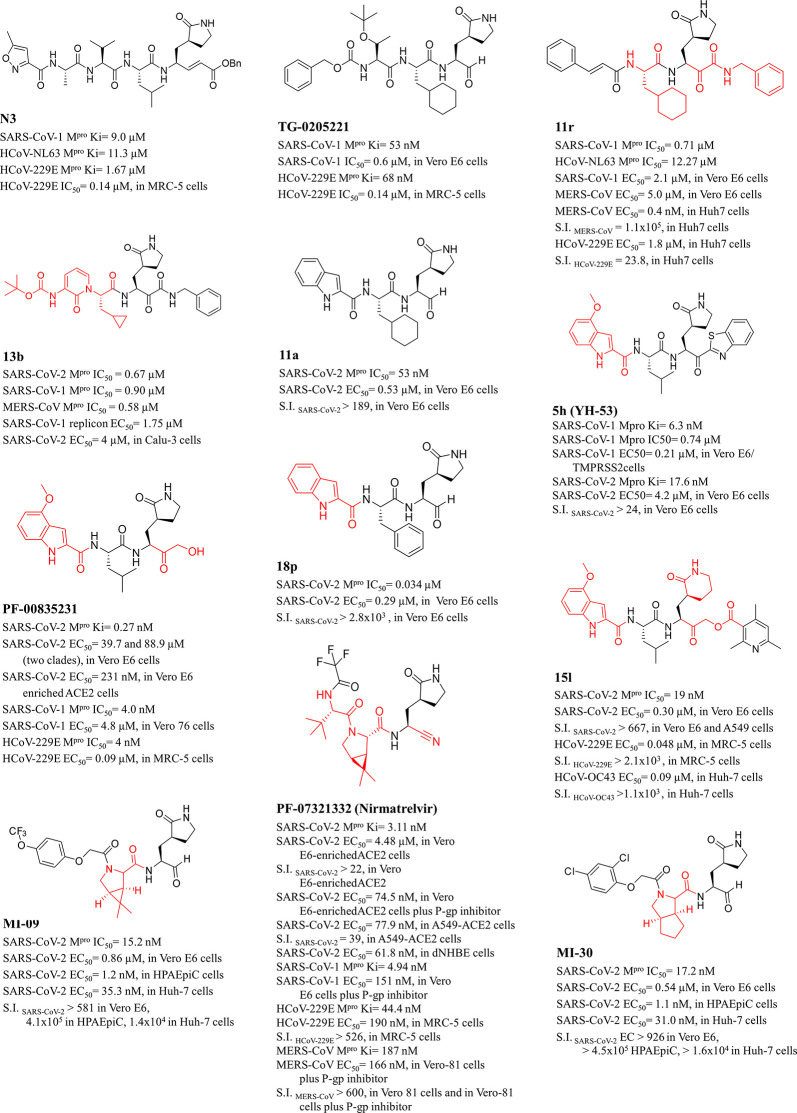
Chemical Structures, Enzyme Inhibition Profile, and Selectivity Index (SI) of Peptidomimetic M^pro^ Inhibitors^a^

a The main fragments explored as innovative P elements with respect to TG-0205221 (template molecule) are highlighted in red.

In the following paragraphs we will describe the efforts made in the past decade to design inhibitors of M^pro^ for both SARS-CoV-1 and SARS-CoV-2 and, finally, we will discuss their repurposing for the current and future outbreaks.

The M^pro^ inhibitor TG-0205221 is here proposed as a template molecule to guide the critical discussion of the main P elements that have been investigated during the drug discovery process since the SARS-CoV-1 outbreak.^27^

**Figure 4:**
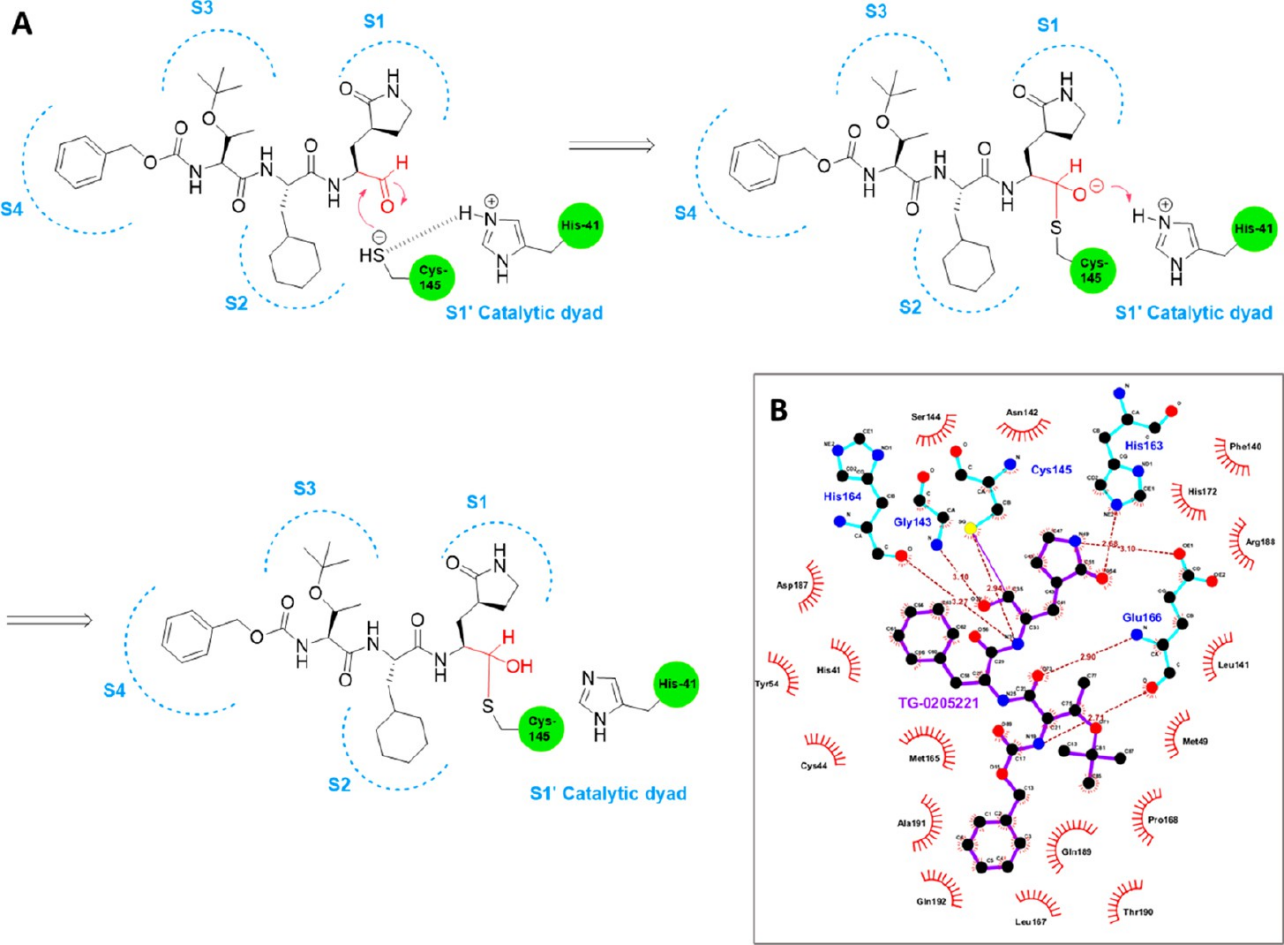
Proposed inactivation mechanism of M^pro^ Cys145 by TG-0205221 (A) and related key contacts within the SARS-CoV-2 active site pocket (PDB ID 7C8T(28)), as shown by a Ligplot diagram (B).

The first M^pro^ inhibitors characteristically incorporated in their structure a substrate-like peptide fragment and a warhead, capable of establishing a covalent bond with the catalytic Cys145 (Figure 4A). As the primary substrate specificity in S1 was a glutamine (Gln) residue, a surrogate was explored in the inhibitor P1 site and, especially, the γ-lactam ring (2-pyrrolidinone ring) emerged as the most suitable moiety.^11^ The P1-Gln specificity is conserved in almost all known human coronavirus M^pro^ cleavage sites^29^ and is considered an absolute requirement for the polyprotein cleavage and a specific property of viral protease.^25^ Genome sequence analysis of HCoV-NL63 and HCoV-HKU1 revealed that in 1 out of 11 M^pro^ cleavage sites histidine replaces glutamine at the P1 position.^30^ Accordingly, Goetz et al. demonstrated that SARS-CoV-1 M^pro^ also recognized P1-His containing substrates with an equivalent *k*_cat_/*K*_M_ as the corresponding P1-Gln substrates; this further P1-His motif, while preserving unaltered the specificity for M^pro^ over 30 host proteases,^31^ opened the way for other types of P1 bioisosteric substitutions in the search for novel M^pro^ inhibitors.

Regarding the reactive warheads, aldehyde, Michael acceptor, halo-methyl ketone, aza-epoxide, aziridine, nitrile, and α-ketoamide functionalities have widely been explored since the first coronavirus outbreak.^32−35^ The enzyme’s inactivation process starts with a noncovalent interaction with the inhibitor that arranges its warhead close to the thiolate anion of the catalytic cysteine, leading to the formation of a covalent adduct. Sometimes the inactivation is irreversible (epoxide, aziridine, halo-methyl ketones, and Michael acceptor groups), whereas compounds bearing an aldehyde or a ketone warhead are reported as reversible inhibitors against SARS-CoV-1 M^pro^. This is due to the lower stability of the hemithio-acetal/ketal adducts which may dissociate from and restore the free form of the enzyme. More interestingly, the reversible warhead upon nucleophilic cysteine attack may result in a different functional group (OH, NH) at the P1 site, forming H-bonds with residues at the bottom of the S1 pocket directly and/or through a water-bridged molecule.^27,31^ Besides the covalent bond interaction, these inhibitors form a high number of H-bonds (7–10) at the S1 site, while numerous hydrophobic contacts are made at S2–S4 sites, altogether concurring to an effective stabilization of the enzyme–inhibitor complex, as exemplified by TG-0205221 (Figure 4B).^27^

At the P2 position leucine is strongly preferred, even if considerable diversity may be tolerated, as observed in the interactions with inhibitors bearing phenylalanine, 4-fluorophenylalanine, methionine, or valine moieties, at the expense of a decrease in the cleavage rate.^31^ Notably, the rigid and planar conformation of the aromatic ring is less favorable to the binding of the S2 hydrophobic pocket, while the more flexible cyclohexylmethyl framework (cyclohexyl-alanine residue in TG-0205221) better fits in a stable chair conformation.^27^ TG-0205221 displayed nanomolar *K*_i_ values against SARS-CoV-1 and human coronavirus 229E (HCoV-229E) M^pro^ enzymes and submicromolar potencies in *in vitro* assays against the respective viruses (Table 1). Such an antiviral profile may also be significant in the context of the COVID-19 pandemic, since SARS-CoV-1, MERS-CoV, and the nonsevere human coronavirus strains 229E, NL-63, and OC43 have consistently drawn attention as models of SARS-CoV-2 for preclinical screening and designing of antivirals. Those reported in Table 1 summarize the knowledge accumulated so far also for other anti-coronaviruses.

The P3 site residue was reported not to be critical for a specific binding and may orient toward the bulk solvent or shift to the P2 site.^11^ The most recurrent motif in this position is a valine residue or its *tert*-butoxy analogue (TG-0205221, Table 1), although the cinnamoyl group is also used by a few peptide inhibitors at the expense of a decrease of at least 1 order of magnitude in their activity.^27^ Diverse series of small alkyls or aryls and heteroaryls, even extended with small alkyl linkers, were investigated as the P4 unit. The benzyloxy carbonyl group (CBZ) proved to be the best group for this site (TG-0205221), being locked in a unique folding conformation which allows the aromatic ring to form strong lipophilic interactions with its environment.^27,31^

**Figure 5:**
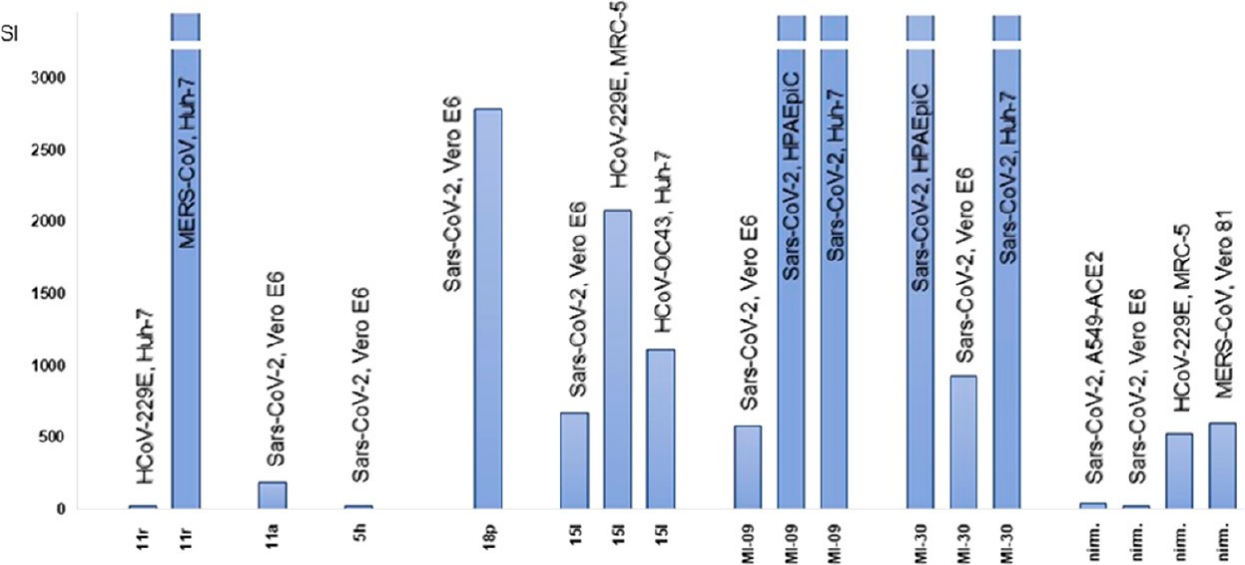
Selectivity indices (as the ratio CC_50_/EC_50_ for each cell line) of the most active peptidomimetics. The SI values herein represented in the *Y* axis up to 3 × 10^3^ reached 10^5^ in the best cases.

**Figure 6:**
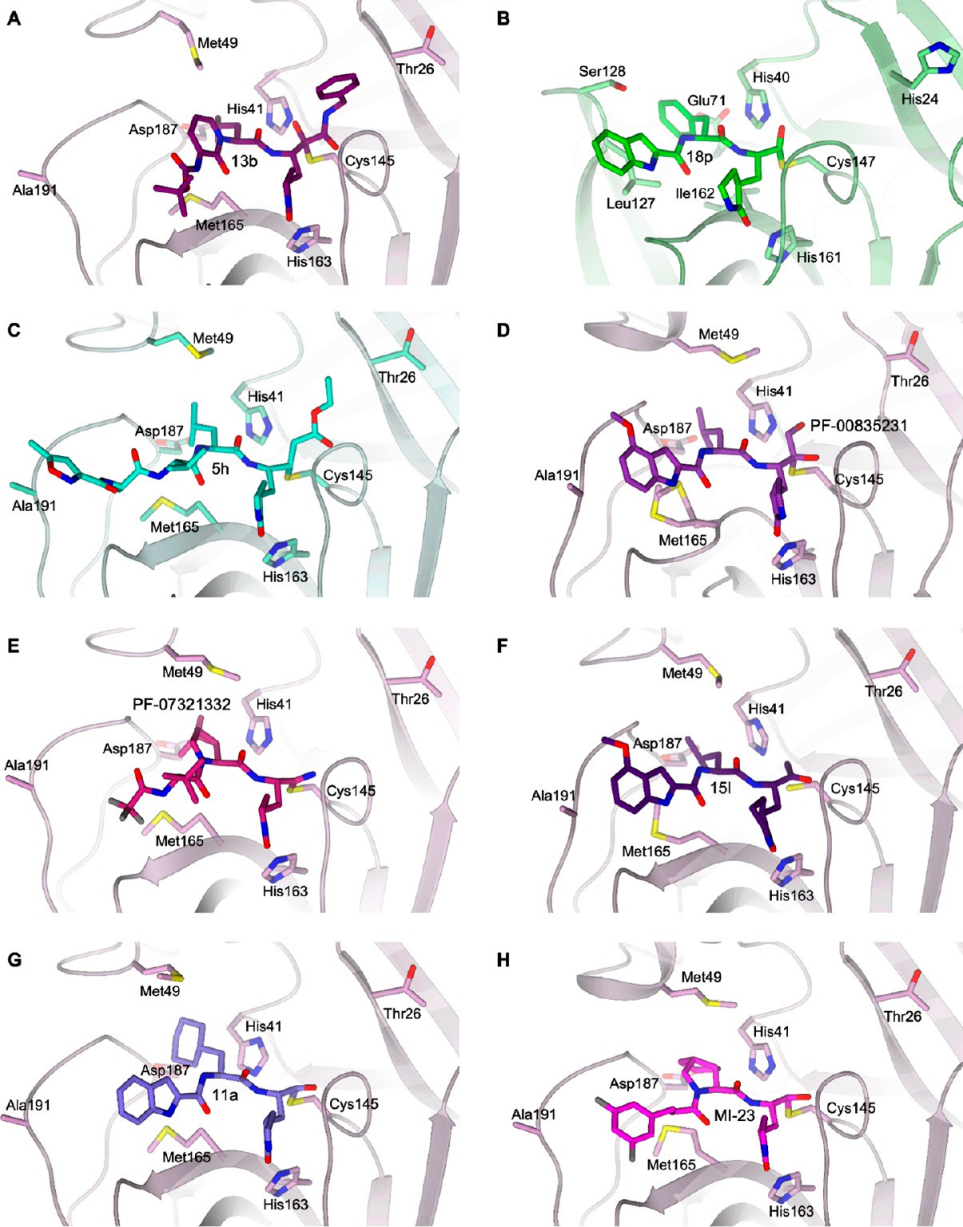
Details of the X-ray crystallographic complexes SARS-CoV-2 M^pro^: inhibitors in the same binding site framework. (A) **13b** (dark violet carbons) in complex with SARS-CoV-2 M^pro^ (lilac, PDB ID 6Y2F(6)). (B) **18p** (green carbons) in complex with EV71 3C^pro^ (light green, PDB ID 7DNC(38)). (C) **5h** (cyan carbons) in complex with SARS-CoV-1 M^pro^ (light green, PDB ID 1WOF(8)). (D) PF-00835231 (purple carbons) in complex with SARS-CoV-2 M^pro^ (lilac, PDB ID 6XHM(42)). (E) PF-07321332 (dark pink carbons) in complex with SARS-CoV-2 M^pro^ (lilac, PDB ID 7VH8(46)). (F) **15l** (dark purple carbons) in complex with SARS-CoV-2 M^pro^ (lilac, PDB ID 7MBI(47)). (G) **11a** (violet carbons) in complex with SARS-CoV-2 M^pro^ (lilac, PDB ID 6LZE(22)). (H) MI-23 (magenta carbons) in complex with SARS-CoV-2 M^pro^ (lilac, PDB ID 7D3I(48)). The protein is shown in a cartoon; the ligand and the residues lining the pocket are shown in capped sticks. The color scheme is red for oxygen, blue for nitrogen, yellow for sulfur, and gray for halogens.

The best examples of peptidomimetics discovered up to now are reported in Table 1 including their *in vitro* biological properties and selectivity index (SI; as the ratio between CC_50_ and EC_50_), which is also depicted in Figure 5 for the purpose of a comparison. These M^pro^ inhibitors demonstrate favorable selectivity index values that, in the best cases, reached 10^5^ (Figure 5). It is worth noting that each compound exhibits a variable SI depending on the different sensitiveness (expressed by the CC_50_ value) of the animal/human cell line used as host to support the replication of coronaviruses. Finally, to integrate the information framework on these compounds, in Figure 6 their binding modes to SARS-CoV-2 M^pro^ are proposed.

A step-by-step analysis of the crystal structure of SARS-CoV-1 M^pro^ in comparison with HCoV-NL63 M^pro^ and CVB3 3C^pro^ in complex with a tripeptide α-ketoamide inhibitor allowed the generation of unprecedented broad spectrum anti-coronavirus and anti-enterovirus inhibitors.^32^ As the SARS-CoV-1 S2 subpocket is larger than those of the other two viruses, it can accommodate molecules bearing P2 groups of variable steric hindrance but endowed with flexibility for a more appropriate binding mode (Figure 2).

Therefore, the flexible and lipophilic cyclopentylmethyl or cyclohexylmethyl groups were identified as the best performing P2 moieties, as experienced by the cyclohexylmethyl α-ketoamide peptide **11r** reaching an outstanding EC_50_ value of 0.4 nM against MERS-CoV in Huh7 cells and SI = 1.1 × 10^5^ (Table 1, Figure 5).^32^

Hilgenfeld et al.^6^ further modified the P3 cinnamoyl moiety of **11r** by a two rounds step optimization, first by incorporation of the P2–P3 amide bond into a pyridone ring to improve the half-life in plasma, followed by the introduction of a Boc group in place of a cinnamoyl moiety to increase the solubility in plasma and to reduce the binding to plasma proteins. Lastly, the P2 cyclohexylmethyl moiety was replaced by the smaller cyclopropylmethyl one, leading to a distinctive pan-coronavirus profile for inhibitor **13b** (Table 1, Figure 6A). This optimized molecule is characterized by a pronounced lung tropism and is suitable for inhalation therapy.^6^**13b** adopts an extended conformation within the SARS-CoV-2 M^pro^ substrate-binding site covering all the key subsites S1–S4 (Figure 6A).

Fragment-based drug discovery (FBDD) strategies^36,37^ were applied for the design of a novel library of peptidomimetic aldehydes, cross-referencing key pharmacophore features extracted from three known EV71 3C^pro^ lead inhibitors.^38^ In particular, the small dipeptidyl derivative **18p** shown in Table 1 proved to be a broad-spectrum anti-EV agent targeting 3C^pro^ by binding to the S1, S2, and S4 subsites (Figure 6B). Due to the structural similarity between the 3C^pro^ of EV71 and that of SARS-CoV-2 M^pro^, both of which have a crucial catalytic dyad composed of a cysteine and a histidine, **18p** was also found active against M^pro^, thus blocking the replication of SARS-CoV-2 with an SI of 2.8 × 10^3^ toward Vero E6 cells (Table 1, Figure 5) and good preliminary PK properties. It is worth noting that the indole ring is frequently proposed as an efficient P3 motif, in diverse examples of M^pro^ inhibitors presented here.^39^ In particular, the presence of the 4-methoxy group on the indole ring improved the antiviral potency profile, as observed for **5h**, whose *K*_i_ values fell in the submicromolar to nanomolar range (**5h**, Table 1, Figure 6C). **5h** forms extensive contacts inside the M^pro^ catalytic cavity, effectively targeting all the key subsites S1′–S4 (Figure 6C). The COVID-19 outbreak has sparked new interest in the potential of compound **5h** (also named YH-53) that has been confirmed to efficiently block SARS-CoV-2 infection, alone and as a combination therapy with remdesivir.^40^ Studies on its pharmacokinetic profile in rats have highlighted issues of low bioavailability due to the metabolic instability of the P1–P2 amide bond, which is vulnerable to hydrolysis reaction.^41^ Due to a growing concern over future drug-resistant variants, an artificial intelligence derived platform analyzed the drug–drug and drug–dose interaction space of six promising experimental or currently deployed therapies, predicting YH-53, nirmatrelvir, and EIDD-1931 (active metabolite of molnupiravir) as the top three-drug combination and the highly synergistic nirmatrelvir interaction with YH-53. These findings were validated by *in vitro* tests against the Omicron variant, suggesting the need for more in-depth preclinical and clinical studies of these combinations of synergistic drugs. Similarly, the ketone-based dipeptide PF-00835231 (Table 1, Figure 6D) was identified as a development candidate for SARS-CoV-1 in 2003, but the success of virus containment measures halted its clinical progress.^42^ Following the COVID-19 pandemic, PF-00835231 has been tested against the novel coronavirus^43^ and was confirmed a potent inhibitor (Table 1) of SARS-CoV-2 M^pro^ thanks to a hydroxymethylketone-driven reversible interaction with the protease active site of which it occupies the S1, S2, and S4 subsites (Figure 6D). This compound demonstrated high selectivity for M^pro^ inhibition over a panel of proteases (IC_50_ proteases > 10 μM) and efficacy in cellular assays against SARS-CoV-1, HCoV 229E, and two different clades of SARS-CoV-2 (Table 1) whose M^pro^ amino acid sequences are identical. Even better, its potency profile against SARS-CoV-2 strains improved from 117- to 173-fold in the presence of a P-gp (MDR1) inhibitor that is able to block the efflux transporter P-gp, overexpressed in monkey Vero E6 cells. Conversely, in human airway cell models the extremely low levels of P-gp do not negatively impact PF-00835231’s performance.^43^ It is worth noting that M^pro^ is also 100% conserved in the SARS-CoV-2 α and γ variants, while β variant carries the amino acid substitution K90R, far from the M^pro^ active site and not expected to influence the substrate/inhibitor specificity.^44^ PF-00835231 also exhibits an additive/synergistic effect in combination with remdesivir against SARS-CoV-2.^43^ PF-00835231, in the form of its more soluble phosphate ester prodrug PF-07304814 (lufotrelvir) for iv treatment, has recently completed a phase 1 trial to evaluate its safety, tolerability, and pharmacokinetics (NCT04535167, results not released yet). Meanwhile, Pfizer was also able to advance an oral SARS-CoV-2 M^pro^ inhibitor (PF-07321332, nirmatrelvir) through multiple rounds of chemical optimization of PF-00835231 for oral bioavailability. The P1′ nitrile warhead, forming a reversible covalent thioimidate adduct with Cys145, and the bicycloproline and 3-methyl-l-valine fragments of the HCV protease inhibitor boceprevir positively contribute to the improvement of its pharmacodynamic and pharmacokinetic profile.^45^ The structural characterization of PF-07321332 in complex with SARS-CoV-2 M^pro^ shows its ability to effectively target all the key subsites S1–S4 (Figure 6E), expanding the binding properties of PF-00835231 (Figure 6D), which fails to occupy the S3 pocket.

PF-07321332 showed *in vitro* pan-human coronavirus antiviral activity in different cellular systems (Table 1, Figure 6E^46^), irrespective of their levels of P-gp expression. In the case of Vero cells (E6 and 81), in contrast to human lung carcinoma A549 and dNHBE (differentiated normal human bronchial epithelial—EpiAirway) cell lines, the codosing with a P-gp inhibitor was necessary to derive more reliable data on its antiviral effectiveness (Table 1, Figure 5). PF-07321332 also displayed excellent off-target selectivity and *in vivo* safety profiles.

With this background, PF-07321332 has entered clinical studies as a combination therapy with the HIV drug ritonavir, able to slow down the metabolism of the protease inhibitor. Their association (Paxlovid) gave in phase II/III trials encouraging results by reducing hospitalization or death by 89% when administered within 3 days of symptom onset.^49^

**Figure 7:**
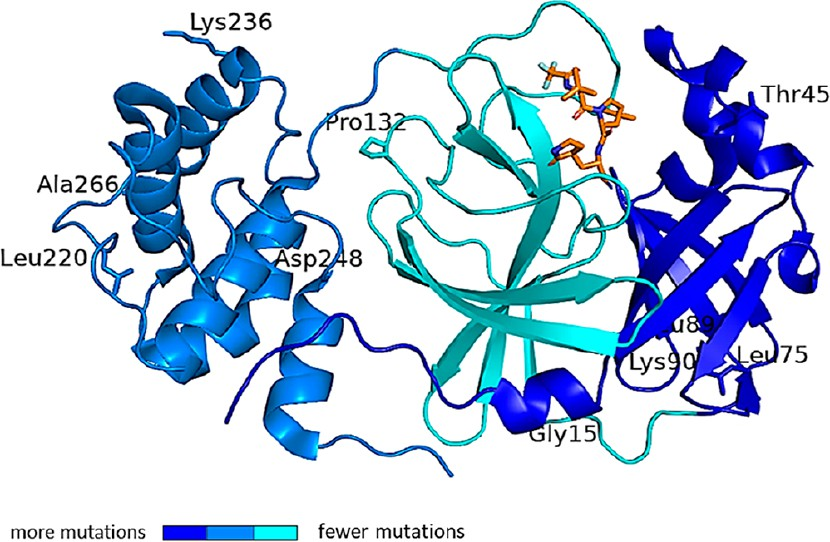
SARS-CoV-2 M^pro^ structure in complex with the drug nirmatrelvir (PDB ID 7VH8(46)), orange sticks. The protein is represented as a blue color code map to evidence the rate of mutation affecting each domain, according to the literature.^50,51^ The rate of mutation was calculated on the basis of the raw number of the registered amino acid variants and represented as color codes (generated with PyMol, Version 2.5.2). The most variable residues are labelled. The drug sits in the binding pocket in domain II, the least affected by mutation frequency.

Comparative analysis of the whole genome of circulating SARS-CoV-2 mutant strains revealed the G15S, K90R, P132H, D248E, T45I, L75F, L89F, L220F, K236R, and A266V mutations are the most frequent (Figure 7).^17,18^ These mutations are not located near the active site of the M^pro^ and no difference in susceptibility for nirmatrelvir has been observed against the M^pro^ of the prevalent VOCs, which carry at varying frequencies the K90R (α, β, γ), G15S (λ), and P132H (ο) mutations.^18^

The X-ray crystal structures of nirmatrelvir bound to K90R, G15S, and P132H M^pro^ variants exhibit a binding mode equivalent to that of the wild-type enzyme, whose conformation is not altered by these mutations.^18^ The reciprocal adaptability of the enzyme to nirmatrelvir may explain why the drug retains its *in vitro* antiviral activity against the Omicron variant relative to wild-type virus.^52,53^ M^pro^ domain II is less prone to critical mutations (Figure 7), strengthening the interest as a valid drug target domain.

Acyloxymethylketone warhead peptidomimetics have been recently proposed as potent inhibitors of the SARS-CoV-2 M^pro^.^47^ On the one hand, different α-acyloxy groups were explored at P1′ with the aim to adjust the p*K*_a_ value of the peptides and balance the excessive reactivity of the warhead in the rapid irreversible adducts which might also sequester glutathione or cause cytotoxicity. On the other, the Gln mimetic γ-lactam P1 motif was enlarged to a six-membered lactam ring, while holding constant the P2-Leu residue and the 4-methoxyindole ring as a key P3 element. Compound **15l**, reported in Table 1 and Figure 6F, ranked a top position for the potent inhibition of SARS-CoV-2 M^pro^ and SARS-CoV-2 replication *in vitro* and excellent plasma stability, despite containing an ester function. Like PF-00835231 (Figure 6D), it effectively targets the S1, S2, and S4 pockets within the SARS-CoV-2 M^pro^ active site (Figure 6F). Good glutathione stability and selectivity for SARS-CoV-2 M^pro^ over cathepsin B and cathepsin S indicated the α-acyloxy warheads as endowed with a discriminant reactivity. Compound **15l** also displayed good antiviral potency against HCoV-229E and HCoV-OC43 viruses (Table 1). However, the metabolic instability and efflux transporter recognition of the compound were correlated to its high lipophilicity, thus suggesting the need for further improvements to obtain more adequate ADME properties.

By analyzing the substrate-binding pocket of SARS-CoV-1 M^pro^, Dai et al.^22^ designed the inhibitor **11a** (Table 1, Figure 6G) characterized by a reactive aldehyde warhead in P1, an (*S*)-γ-lactam ring that occupies the S1 site of M^pro^, a recurrent cyclohexyl moiety into P2 inside the S2 pocket, and again the indole group into P3 to form new hydrogen bonds with S4 and improve drug-like properties. This compound showed a high SARS-CoV-2 M^pro^ inhibition potency and a good antiviral activity in cell culture. *In vitro* and *in vivo* studies revealed no obvious toxicity and a good PK profile, suggesting **11a** as a valuable drug candidate for clinical evaluation.^22^

The analysis of the S4 site of SARS-CoV-2 M^pro^ allowed Qiao et al. to develop covalent-bonding M^pro^ aldehyde dipeptidyl inhibitors, incorporating hydrophobic subgroups of medium size to intercept P3 and enhance their potency and PK properties.^48^ The rigid and hydrophobic bicycloproline ring, derived from the antiviral drugs boceprevir and telaprevir,^54^ respectively, was successfully explored. In particular, compounds MI-09 and MI-30 (Table 1; see also MI-23 analogue in Figure 6H, targeting the S1, S2, and S4 subsites within the SARS-CoV-2 M^pro^ catalytic cavity) exhibited potent *in vitro* and *in vivo* antiviral activity, significantly reducing lung viral loads and lung lesions in a transgenic mouse model of SARS-CoV-2 infection.^48^ Both also displayed good pharmacokinetic and safety profiles *in vitro* (Figure 5) and *in vivo*, thus representing a valuable starting point toward the development of orally available anti-SARS-CoV-2 drugs.

### Nonpeptidomimetic Inhibitors of M^pro^ Catalytic Site

The development of M^pro^ inhibitors also turned to nonpeptidic small molecules expected to have more suitable pharmacokinetic profiles (due to their lower molecular weights, higher membrane permeabilities, longer half-lives) and, potentially, lower production costs.^11^

The serotonin antagonist cinanserin was one of the first nonpeptide-based compounds identified by a virtual screening program which displayed the capacity of irreversibly reacting with Cys145 through the double bond of the cinnamyl amide warhead (IC_50_ value of ∼5 μM). The antiviral assays confirmed the inhibition of SARS-CoV-1 and HCoV-229E virus strains with IC_50_ values of 34 and 19 μM, respectively.^55^

As a valuable option, warhead-based small molecule inhibitors bearing reactive esters and ketone moieties were previously reviewed.^11,56^ The most notable chemical structures were the heteroaromatic esters of 3-hydroxypyridine which drew the attention of different research groups as M^pro^ inhibitors.^57,58^ The mode of action involves the irreversible acylation of the active site Cys145 by means of the ester bridge.^57^ While the halopyridine moiety of **39** (Table 2) and analogues fits comfortably in the S1 substrate binding site, establishing prevalently van der Waals contacts, the furan ring is located near the catalytic residue Cys145, forming hydrophobic contacts.^57,59^

**Table 2:**
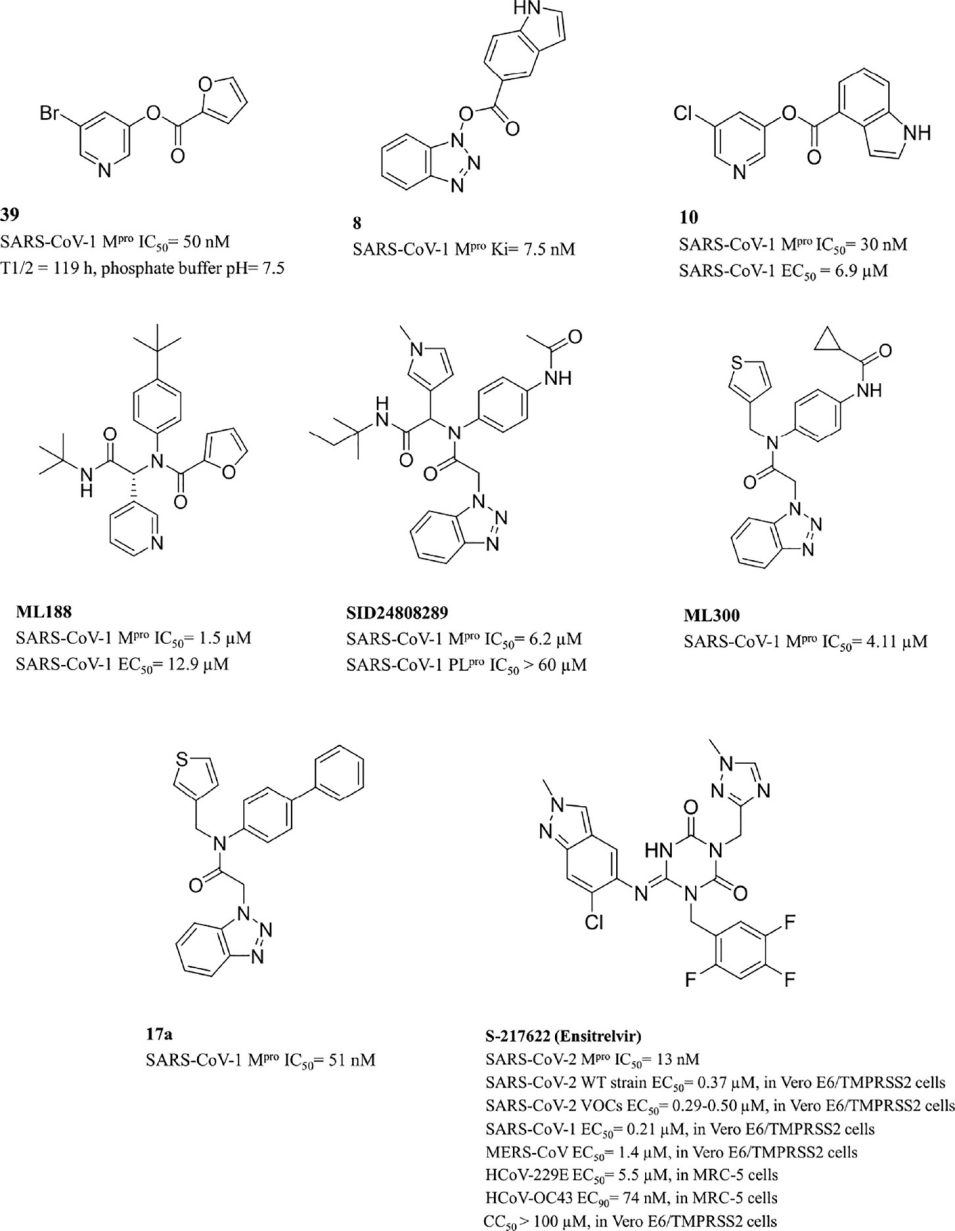
Chemical Structures, Enzyme Inhibition Data, and Anti-Coronavirus Activity of Nonpeptidomimetic M^pro^ Inhibitors

Wu et al.^60^ described a new class of benzotriazole ester based M^pro^ inhibitors where HOBt resembled the previously described pyridine moiety and formed an ester linkage to anthranilic acid and related structures, where the amino group was shifted from the *ortho* to the *para* position or was incorporated into an indole or a benzimidazole ring. Compound **8** (Table 2) was found to be the most potent M^pro^ inactivator. This class of compounds shares a similar mechanism of action and interaction pattern of halopyridine esters. Despite their very impressive SARS-CoV-1 M^pro^ inhibitory potency, their related antiviral activity is poor or totally absent.

Mesecar et al.^58^ merged the key units identified in previous studies as the 5-chloro-3-hydroxypyridine moiety and the indole ring, by means of an ester function, whose position on the indole ring was found to be critical to the potency profile, as exemplified by the most effective 4-substituted derivative **10** (Table 2).

Since covalently bound inhibitors may be impaired by a high risk for off-target side effects and toxicity,^61^ researchers also focused on the design of noncovalent binders. Following the first coronavirus outbreak, different main core structures were proposed as noncovalent M^pro^ inhibitors, such as bifunctional boronic acids, metal-conjugated compounds and pyrazolone derivatives, and others.^11^

**Figure 8:**
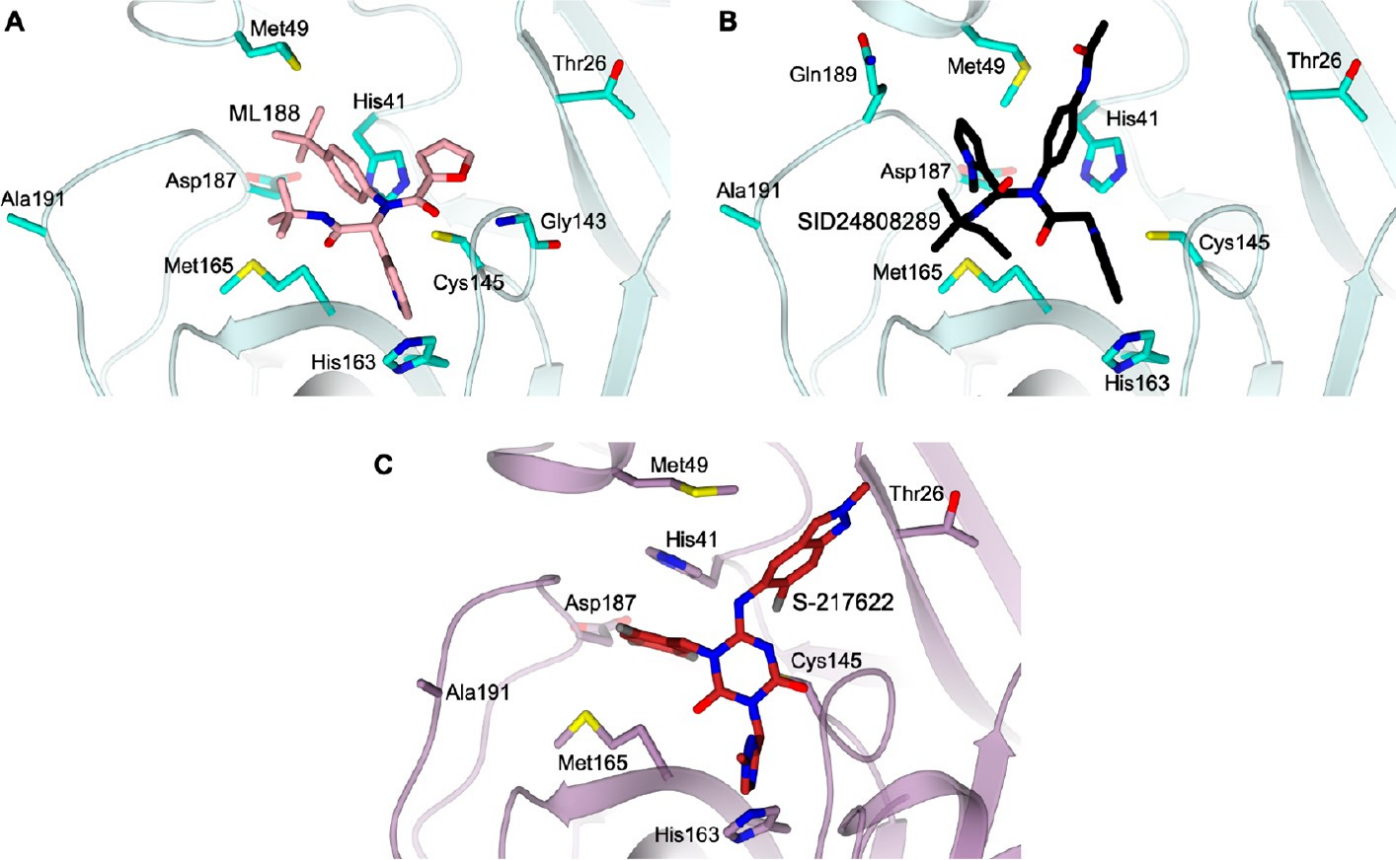
Active site views of the structures of (A, B) SARS-CoV-1 M^pro^ (cyan) in complex with (A) ML188 (pink carbons, PDB ID 3V3M(55)) and (B) SID24808289 (black carbons, PDB ID 4MDS(61)) and (C) SARS-CoV-2 M^pro^ (lilac) in complex with S-217622 (firebrick carbons, PDB ID 7VU6(78)). The protein is shown in a cartoon; the ligand and the residues lining the pocket are shown in capped sticks. Oxygen atoms are colored red, nitrogen atoms are blue, sulfur atoms are yellow, and halogen atoms are gray.

Worthy of note is the class of furyl amides^56^ that were identified from multiple rounds of screening in HTS technology of a large NIH compounds library. Subsequent hit optimization studies explored the chemical space around the P2, P1, and P1′ moieties of the furan amide scaffold. The *R*-enantiomer ML188 resulted as the best performing inhibitor (Table 2), with a highly selective profile for M^pro^, with respect to papain-like protease (PL^pro^), and 68 G protein coupled receptors (GPCRs), ion channels, and transporters. As the PL^pro^ tertiary structure is remarkably similar to that of cellular ubiquitin specific proteases (USPs), to reduce the chance of provoking off-target activity, it is of the utmost importance that inhibitors be made more selective for M^pro^ than for PL^pro^. ML188 accommodates the substrate subpockets in the enzyme active site traditionally occupied by peptidomimetics, while the furan ring oxygen and the amide carbonyl oxygen make a bifurcated interaction with the backbone NH of Gly143 (Figure 8A). On the basis of this key contact, the authors^56^ prepared a chemical library manipulating exclusively the P1′ motif of ML188, without observing any improvement over the prototype, which remained unsurpassed for potency. The same authors also investigated benzotriazole derivatives as noncovalent inhibitors^62^ and observed a different binding mode with respect to ML188. The lead molecule SID24808289 caused an induced-fit reorganization of the Gln189 and Met49 side chain rotamers within S2–S4 and S2–S1′ pockets, respectively, as imposed by its *N*-methyl pyrrole and the *N*-acetylanilide moieties (Figure 8B).

After the *N*-methyl pyrrole was replaced with an equipotent 3-thienyl moiety, as a second step, the acetamide group in the P2–P1′ region was substituted with branched isopropyl and cyclobutyl moieties, which led only to slight increases in potency. The limited success from the above studies prompted a scaffold simplification strategy, in which the lipophilic P3 motif, being unfavorably solvent exposed in ML188, was removed. From this, ML300 (Table 2) emerged as the most promising compound, by virtue of its valuable M^pro^ inhibitory activity and the significant improvement of its lipophilic ligand efficiency (LLE) value over those of the parent compounds. The attempt to further ameliorate the PK and potency profiles contributed to the discovery of the biaryl moiety as a more efficient P2–P1′ substitution (**17a**, Table 2). However, protein flexibility and induced fit strongly limited the application of structure-based *in silico* simulations. The concept of flexibility has frequently been pointed out also for SARS-CoV-2 M^pro^ and could undermine the repurposing of the aforementioned inhibitors toward other coronavirus M^pro^s.^63^ Despite these observations, several virtual screening campaigns were conducted, with the aim of identifying suitable new scaffolds to target M^pro^.^64−67^ Apart from the most used databases in virtual screenings, such as ZINC, ChEMBL, and Pubchem, different small molecule sources were considered, like the traditional Chinese medicine database,^68^ Indian spices,^69^ phytochemical libraries,^70^ or marine natural products.^71^ Approved antivirals were also tested as possible M^pro^ inhibitors, with the aim of repurposing molecules that had already undergone clinical trials. The combination of the anti-HIV drug lopinavir in association with ritonavir (Kaletra) displayed *in vitro* anti-CoV activity^72^ and reduced adverse effects when administered in association with ribavirin.^1^ To date, no positive outcome has been observed with lopinavir–ritonavir treatment in adults hospitalized with severe COVID-19.^73^ Further clinical trials, started in 2020, may help to confirm or exclude the possibility of a treatment benefit, either as a monotherapy [ClinicalTrials.gov: NCT04372628], or as a combination regimen with hydroxychloroquine [ClinicalTrials.gov: NCT04376814] or remdesivir [ClinicalTrials.gov: NCT04738045]. Similarly, different repurposing studies were carried out *in silico*, since the first crystallographic structure of SARS-CoV-2 M^pro^ was released. To mention only some of them, Wang performed virtual screening, molecular dynamics simulations, and MM-PBSA-WSAS energy calculations for approved drugs and drug candidates toward M^pro^, finding carfilzomib, eravacycline, valrubicin, lopinavir, and elbasivir as promising candidates.^74^ Lopinavir, ritonavir, darunavir, and cobicistat were also found by Pant et al. applying a similar procedure.^75^ In one of the first virtual screenings, Chen et al. identified, among the Drugs-lib data set of purchasable drugs, ledipasvir and velpatasvir (used to treat hepatitis C virus (HCV)) as possible therapeutic agents with minimal side effects.^76^ Another study identified talampicillin (a prodrug of ampicillin) and lurasidone (an antipsychotic for schizophrenia treatment) as possible M^pro^ inhibitors;^77^ indeed, many other *in silico* studies have been performed.^33,64^

In 2022, by applying a docking-based virtual screening of an in-house compound library using the crystal structures of the M^pro^ and ML188-like noncovalent small molecules (PDB ID 6W63(79)), Unoh et al. reported the development of a nonpeptidic, noncovalent orally active drug candidate, S-217622, targeting SARS-CoV-2 M^pro^^78^ (Table 2, Figure 8C). This compound showed high selectivity for SARS-CoV-2 M^pro^ over a panel of host human proteases and a high metabolic stability to CYP450 3A4, thus not requiring the coadministration of ritonavir as is the case with its competitor, the peptidomimetic nirmatrelvir (Paxlovid formulation). Thanks to its broad-spectrum anti-coronavirus activity, including VOCs (EC_50_ = 0.29–0.50 μM, Table 2) and favorable preclinical profile, S-217622 rapidly progressed through clinical trials to phase 3 studies (clinical trial registration no. jRCT2031210350) as a once-daily oral therapeutic agent for COVID-19.^80,81^ Through molecular dynamic simulations, Xiong et al.^82^ elucidated its molecular mechanism of SARS-CoV-2 M^pro^ inhibition, observing a difference in the movement modes between the S-217622–M^pro^ complex and apoenzyme. S-217622 was shown to inhibit the motility intensity of M^pro^ stabilizing the binding with the target, thanks to multiple hydrogen bonds and hydrophobic interactions with a hot-spot signature lined by His41, Met165, Cys145, Glu166, and His163. Interestingly, from the *in silico* analysis of the resistance of S-217622 to VOCs, no significant differences in the interaction pattern were observed; thus the drug candidate was predicted to efficiently target mainstream variants as well as wild-type M^pro^s, in agreement with the above-mentioned experimental findings.

The recent emergence of the SARS-CoV-2 Omicron variants (B.1.1.529 lineage) exhibiting numerous mutations has raised concerns of limited efficacy of current vaccines and therapeutics for COVID-19.^83^ Encouragingly, S-217622, nirmatrelvir, and molnupiravir were verified in rodent models as valuable inhibitors of original Omicron BA.1 and now prevailing BA.2 sublineages. While nirmatrelvir and molnupiravir only reduced the lung virus titers, the treatment with S-217622 (ensitrelvir) additionally decreased the virus titers in the nasal turbinates.^84^ These remarkable results advocate the nomination of ensitrelvir as a prospective oral therapeutic option for COVID-19.

### Allosteric Inhibitors of M^pro^

Through a large-scale X-ray crystallographic screening of M^pro^ against two repurposing libraries, some novel inhibitors were identified. Worthy of note are calpeptin (EC_50_ = 72 nM, CC_50_ > 100 μM) occupying the S1–S3 subpockets of M^pro^ active site like the peptidomimetic inhibitors (N3 in Figure 3A and **13b** in Figure 6A)^33,35^ and pelitinib (EC_50_= 1.25 μM, CC_50_= 13.96 μM), targeting an allosteric binding site featured by a hydrophobic pocket formed by Ile213, Leu253, Gln256, Val297, and Cys300 within the C-terminal domain III.^85^ More interestingly, despite pelitinib being designed as a Michael acceptor inhibitor for an anticancer purpose,^86^ no evidence of its covalent binding to M^pro^ Cys145 was observed.

As mentioned earlier, one of the key points making M^pro^ a druggable target is the fact that this enzyme shares 96% similarity with the same protease in SARS-CoV-1 and 100% identity in the catalytic domain that carries out protein cleavage.^19^

In general, M^pro^ enzymes from different human and animal CoVs are known to display significant homology in both primary amino acid sequence and 3D architecture, providing a strong structural basis for the possible design of pan-coronavirus inhibitors.^25^ However, it has recently been pointed out that, despite the high sequence similarity, SARS-CoV-1 and SARS-CoV-2 M^pro^s exhibit major differences in terms of binding site shape, size, and flexibility, which could jeopardize the repurposing of available drugs. Only 12 residues differ in SARS-CoV-2 M^pro^ with respect to SARS-CoV-1, and only one, Ser46, is in the active site (Figure 1). More specifically, Bzówka et al. compared the dynamics and properties of the two M^pro^ binding sites, by means of classical and mixed-solvent molecular dynamics simulations as well as by evolutionary and stability analysis.^87^ First, the maximal accessible volume (MAW) is quite different: as both proteases significantly reduce the site MAW upon ligand binding, that of SARS-CoV-1 M^pro^ is over 50% larger. Second, the movement of the loops lining the binding site and regulating its accessibility is different. In particular, the C44–P52 loop is more flexible in SARS-CoV-1 than in SARS-CoV-2 M^pro^. Similar conclusions have also been drawn by Gossen et al., who suggested that drug repurposing among SARS-CoV-1 and SARS-CoV-2 inhibitors may not be so straightforward.^63^ In general, the high flexibility of the binding site represents an obstacle for virtual screening campaigns^88^ and could explain why many potential SARS-CoV-2 inhibitors did not reach the clinical trial stage. Mutations occurring at flexible regions could also significantly change the affinity of inhibitors toward M^pro^ and reduce the potential use of this protein as a target for coronavirus treatment. However, residues predicted as not prone to mutation could also provide an anchor for the design of effective drugs.^87^ Regions other than the binding site, such as the space between domains II and III, which contribute to the dimer formation, could also be targeted. In general, the enzymatic activity of M^pro^ depends on the architecture of the active site, which critically stems from the dimerization of the enzyme and the appropriate orientation of each subdomain. This could allow ligands that bind outside of the active site to dampen the enzyme activity,^85^ as experienced by the repurposed drug pelitinib, described above.

### SL Strategies Applied to M^pro^ Inhibitors

In a first step, a strategy based on invariant amino acids can be considered since the number of mutations per position for M^pro^ is still low. M^pro^ is subject to very strong global selection pressure in human cells, and an in-depth study of its future mutational landscape suggests that mutations will appear rapidly.^89^ On the other hand, other viral proteases have acquired many mutations without losing their activity. The HIV protease is a very good example. An increased number of selected mutations is therefore a very likely hypothesis, and SL strategies will therefore have to be considered rapidly.

As M^pro^ comes into contact with other viral and host proteins, the three SL strategies (intragenic, extended intragenic, and intergenic) can be applied to this protein. The SL intragenic strategy (see the method in Perspective and Foreseen Studies: Alliance between Drug Discovery and Genetics) will determine whether the active site is a target for little or no therapeutic escape. It will also determine whether other targets outside the active site would control escape. The extended intragenic SL strategy will allow searching for targets at the interface between M^pro^ and its viral protein substrates since it is responsible for cleaving most of structural and nonstructural SARS-CoV-2 proteins.

**Figure 9:**
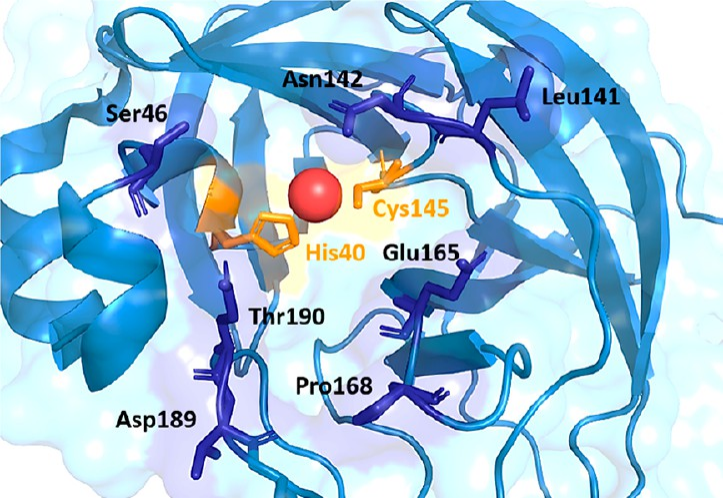
Focus on the catalytic groove of nsp5 protease that accommodates the substrates, occupied by a zinc atom (red dot). His40 and Cys145 constitute the catalytic dyad of the hydrolase which cleaves TMRT1. PDB ID 7NWX.^262^

Finally, regarding the intergenic SL strategy, at least two avenues are conceivable: M^pro^ interacts at least with two cellular proteins, HDAC2 and TRMT1. Wild-type HDAC2 is translated into the cytoplasm and must be transported to the nucleus to act on histones. A M^pro^ cleavage site of HDAC2 predicted by Gordon et al.^13^ seems to be located between the HDAC2 domain and the nuclear localization sequence. HDAC2 has been described to be involved in inflammation and interferon response.^90,91^ Mast et al.^92^ suggested that one can block viral development by drug targeting their synthetic lethal partner. The function of HDAC2 is redundant with the function of HDAC1,^93^ and the HDAC1 and HDAC2 double mutant seems to have a very decreased cell proliferation. Thus, the intergenic SL strategy could be applied. During viral infection, M^pro^ proteolyzes HDAC2 thus making it inoperative. In parallel, if another drug blocks HDAC1, the infected cells could no longer multiply, since the function of HDAC2 would be blocked by the virus and that of HDAC1 by the drug, while in uninfected cells the function of the HDACs would be carried by HDAC2 and thus these cells could grow normally. The same scenario can be applied to the M^pro^–TRMT1 pair since Gordon et al.^13^ also predicted that M^pro^, whose catalytic pocket is represented in Figure 9, would cleave through its catalytic His–Cys dyad the zinc finger of TRMT1, which is necessary for its nuclear localization signal. Thus, a new drug blocking a possible synthetic lethal partner of TRMT1 could stop the SARS-CoV-2 progression. Interestingly, possible mutations in M^pro^ that would prevent HDAC2 or TRMT1 proteolysis, thus bypassing this strategy, would affect its functionality rendering the virus almost certainly nonreplicative.

## Spike Protein

### Membrane Fusion Inhibitors: Peptides That Bind to Six Helix Bundle Domains

**Figure 10:**
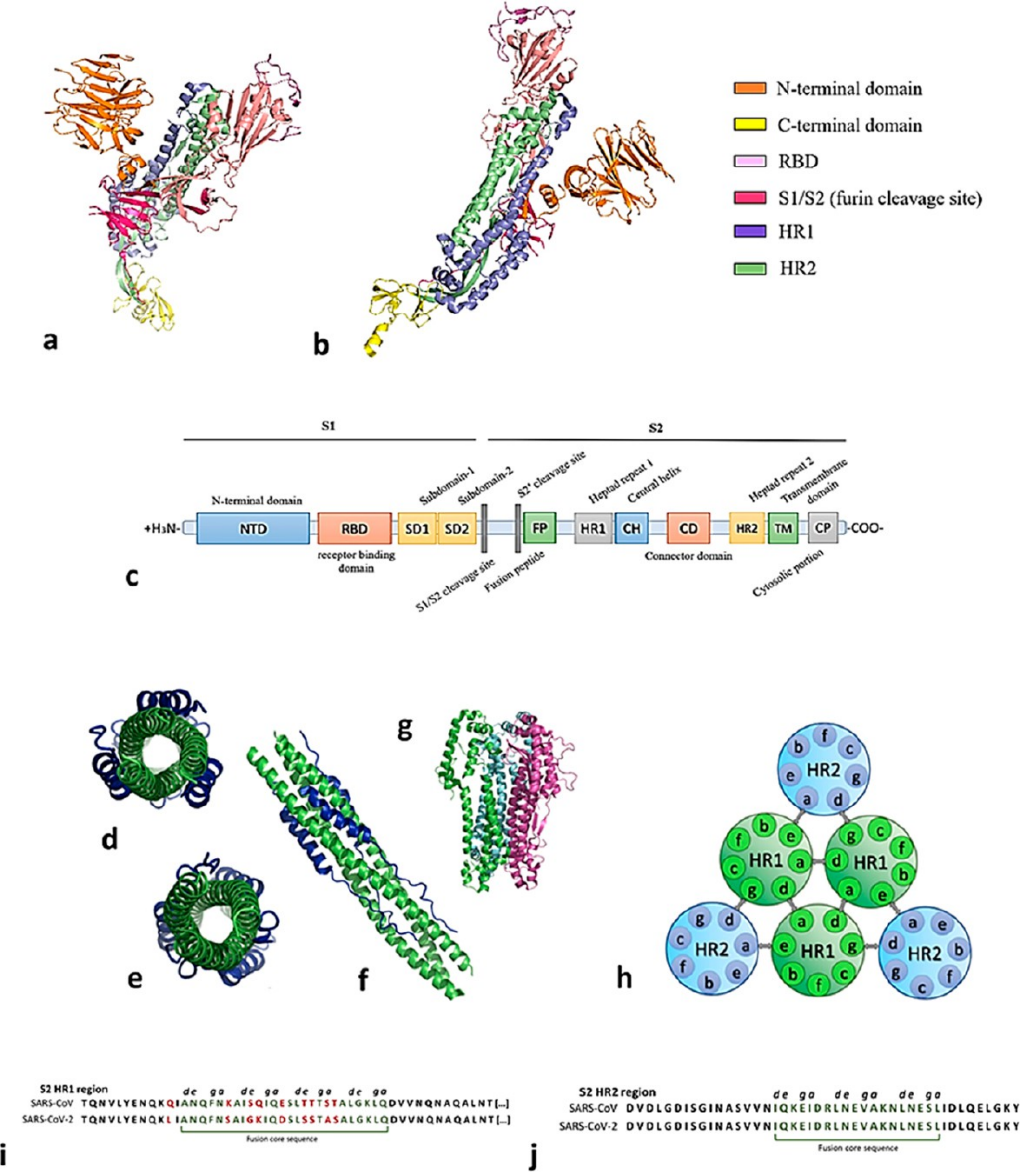
(a–c) Representation of the S glycoprotein main domains. (d) Six helix bundle (6HB) in the heptad repeats region forming contacts to S1. (e) 6HB region from the inner region. (f) 6HB full length (PDB ID 7VX1). (g) α-Helix region of S2 glycoprotein [PDB ID 7TGY). (h) Topological representation of a 6HB domain: *a*–*d*, amino acid residue interactions hold together the internal HR1 core; *d*–*g* and *a*–*e* participate in the interhelical knob-in-hole packing. (i, j) HR1 region shows 92.6% homology between SARS-CoV-1 and SARS-CoV-2, while HR2 shows 100% homology; fusion core sequences are evidenced in dark green and amino acid mutations are shown in red.

The spike protein of β-coronaviruses, a large integral membrane glycoprotein (>1000 amino acids (aa)), has been studied as a possible drug target since the outcome of SARS-CoV-1 epidemics as a strategy to inhibit early phase entry process and membrane fusion. Its sequence can be divided into two main subunits, the first one of which (S1) encloses its N-terminal domain (NTD), mediating the attachment of the virus to the host, the receptor-binding domain (RBD) responsible for SARS-CoV-2 recognition of the target cell, and two additional subdomains (S1/S2). This is separated from the second subunit (S2) composed of a fusion peptide (FP), the N-terminal heptad repeat (HR1), the C-terminal heptad repeat (HR2), a transmembrane (TM) domain, and the cytoplasmic tail (CP), as represented in Figure 10a–c.^94^ After ACE2 recognition and RBD attachment, a massive conformational rearrangement is triggered in the FP, leading to its insertion into the cell membrane by host–guest bilayer fusion. S1 is cleaved from the entire glycoprotein (viral priming process) on a polybasic motif between S1/S2 (furin cleavage site) by the host proteases, and the penetration of S2 into the host cell is enhanced by the rearrangement of the two heptad repeats (HR1 and HR2).

Since the six helix bundle (6-HB) domain in the spike S2 subunit is a highly conserved region situated between the HR1 and HR2 α-helices (Figure 10c), the inhibition of HR1 through a synthetic HR2-mimetic peptide has been the most surveyed and straightforward strategy to target the S protein since the early outcome of SARS-CoV-1.^95^ The dimerization between synthetic peptides and the 6-HB domain prevents it from switching to the active conformation needed for membrane fusion, thus blocking the viral entry. Hereby, the choice of preferring HR2-like peptides in place of HR1-like peptides for the protein inhibition is based on the relative shortness of the former helices (about 4.5 turns versus 21 turns), and was highlighted in a study on their intrinsic stability, α-helicity, and solubility.^96^

The strategy of using fusion-inhibiting peptides against β-coronaviruses was first described at the end of SARS-CoV-1 pandemic, when 36–40 amino acid synthetic peptides (namely “NP” and “CP” series) were designed to target their paralleling HR sequences. The already existing enfuvirtide model peptide (T20), with SJ-2176, which acts with the same mechanism against the 6-HB domain of HIV-1 gp41, was the driving force toward this pharmaceutical approach.^97^ Circular dichroism (CD) and thermal stability (*T*_m_) were used as the main assays to assess synthetic peptides’ α-helicity, their temperature-driven conformational changes, and their ability to dock the viral S2 coiled-coil region.

CP-1 (4.1 kDa, 37 aa) structure and folding was established through surface plasmon resonance (SPR) and CD as a promising model to design fusion inhibitors on the kidney Vero E6 *in vitro* model (EC_50_ = 19 μM). Moreover, when CP-1 and NP-1, its respective homologue targeting HR2, are incubated in equimolar concentrations, a six helix bundle heterocomplex is formed, which folds like the fusogenic core structure of HIV-1 gp41, that could be adapted to the SARS-CoV-2 HR1 region.^98^ Besides the HIV studies, within 2009 four more HR2-mimetic peptides, sHR2-1, sHR2-2, sHR2-8, and sHR2-9, were tested, reaching an EC_50_ of 17 μM, along with 20 other peptides already synthesized.^99^ This engagement supported the study of small peptides inhibiting virus entry. Indeed, a library of 20 recombinant peptides derived from the HR2 amino acid sequences, namely HR2-1 to HR2-20, were tested. HR2-18 (sequence IQKEIDRLNEVAKNLNESLIDLQELGK, interacting with amino acids 1161–1187 of the HR1 domain) showed an optimal length to inhibit SARS-CoV-1 *in vitro* with an EC_50_ of 1.19 μM for the pseudotyped HIV-luc/SARS virus and 5.2 μM against wild-type SARS-CoV-1,^100^ which further became the models to template MERS and SARS-CoV-2 mimicking peptides.^101^

Lu et al.^101^ reassessed two series of HR-mimetic peptides as an approach to coronaviruses. HR1P (MW 4.47 kDa, 42 aa) and HR2P (MW 4.14 kDa, 36 aa), spanning residues 998–1039 of the HR1 domain and 1251–1286 in the HR2 domain, were expressed as recombinant proteins with the aim to target the amino acid residues within the *a* and *d* positions inside the inner core of the 6HB motif (Figure 10d–j), which are represented by nonpolar residues of Ala, Leu, and Ile and polar residues of Glu, Asp, and Ser; these residues contact one another and stabilize the inner core of the HR1 complex in its active conformation. Indeed, MD showed that the interaction between HR2 and the two neighboring HR1 α-helices involves 15 H-bonds, a hydrophobic groove, and a few salt bridges.

Starting from the above-mentioned peptides, shorter (HR*n*S) and longer (HR*n*L) peptides containing HR cores were tested to determine their inhibition levels by pairing one to another. Results showed that, despite predicted α-helicity, the highest score was gained by the complexes HR1L–HR2P (84.7% α-helicity) and HR1L–HR2L, confirming that the longer the chain, the more stable are the interactions.^102^ Both complexes showed a *T*_m_ of >99 °C. HR2P has IC_50_ = 0.5 μM in cell–cell fusion assay inhibition test and IC_50_ ∼ 0.6 μM in inhibiting the infection of Vero E6 by MERS-CoV, but no inhibition on SARS-CoV-1 infection was measured.^101^ On HR1 of MERS-CoV, the residues Q1020-D1024 form a hydrogen bond, which does not occur in SARS-CoV-1 corresponding residues G928-D932. Furthermore, binding between D1161-T1263 and E1276-K1172 on the HR2 domain of MERS-CoV is stronger than that between Q1161-N1159 and E1276-K1172 of SARS-CoV-1, and a hydrogen bond between Q1009 and Y1280 (HR2) in MERS-CoV is formed, while SARS-CoV-1 lacks the H-acceptor. Those differences are assumed to be responsible for HR2P’s activity on MERS-CoV S protein rather than on that of SARS-CoV-1.^103^ Overall, the small homology in the amino acid ratio from SARS-CoV-1 to MERS-CoV between HR1 and HR2, 56.3 and 33%, respectively, has decreased the interest in the development of a common peptide inhibitor candidate.

The insertion of Glu (E) and Lys (K) residues with a fixed pattern of [*n* + 4 or 3*n*(K/E)] arrangements, obtaining HR2P-M1 and HR2P-M2 (sequence SLTQINTTLLDLEYEMKKLEEVVKKLEESYIDLKEL) peptides, increased the α-helicity from 18.7% to, respectively, 36.4 and 42.4%, the *T*_m_ value, solubility up to 1.8-fold, and cell–cell fusion inhibition on MERS-CoV up to 96% (IC_50_ ∼ 0.55 μM on infection assays using Calu-3 and Vero E6 cells), due to more salt bridge formation between coiled-coil motifs, as previously confirmed on the HIV-1 approach.^104^ Furthermore, intranasal administration of HR2P-M2 fully protected transgenic mice after MERS-CoV exposure, and combination with interferon-β therapy reduced 1000-fold virus titer in lungs.^105^

### Entry Inhibitors: Stapled, Lipoylated, and PEG-Stabilized Peptides

To raise α-helicity and increase stability, also a stapling strategy was adopted. Chemical stapling consists in introducing a carbon chain based ligand covalently bound to two amino acid residues of the synthetic α-helix. Synthetic olefin terminated amino acids were introduced onto *a* and *d* side chains, so that interhelix interactions were not disrupted, and made of (*S*)-2-(4-pentenyl)alanine at *i* and [*i*(aa) + 4] positions. Acquired CD spectra confirmed that P21S8 (sequence LDLTYEMLSLQxVVKxLNESY; “x” indicates stapling positions) and P21S10 (sequence LDLTYEMLSLQQVVKxLNExY) displayed, respectively, 54 and 47% α-helicity, and EC_50_ values of 3.03 and 0.97 μM in inhibition of wild-type MERS-CoV infection to Huh-7 and Calu-3 cells, respectively. They also revealed longer half-lives in rats compared to nonstapled peptide HR2P-M2, an AUC_0–*t*_ 13–27-fold higher in intranasal administration to mice.^106^

A few years ago, before the SARS-CoV-2 outbreak, the organic chemistry of peptides approached the synthesis of lipopeptides, synthetic peptides conjugated to long chain saturated fatty acids. A library of 12 lipopeptides with a palmitoyl residue and an Ac-(X_*a*_E_*b*_E_*c*_X_*d*_Z_*e*_K_*f*_K_*g*_)_5_-β-Ala-K(C16)-NH_2_ motif was tested on Huh-7 cells against MERS-CoV. The peptides LLS (sequence LEELSKKLEELSKKLEELSKKLEELSKKLEELSKK-βA-K (C16) and IIS (sequence IEEISKKIEEISKKIEEISKKIEEISKKIEEISKK-βA-K (C16) showed EC_50_ values of 0.24 and 0.1 μM, respectively.^107^

**Figure 11:**
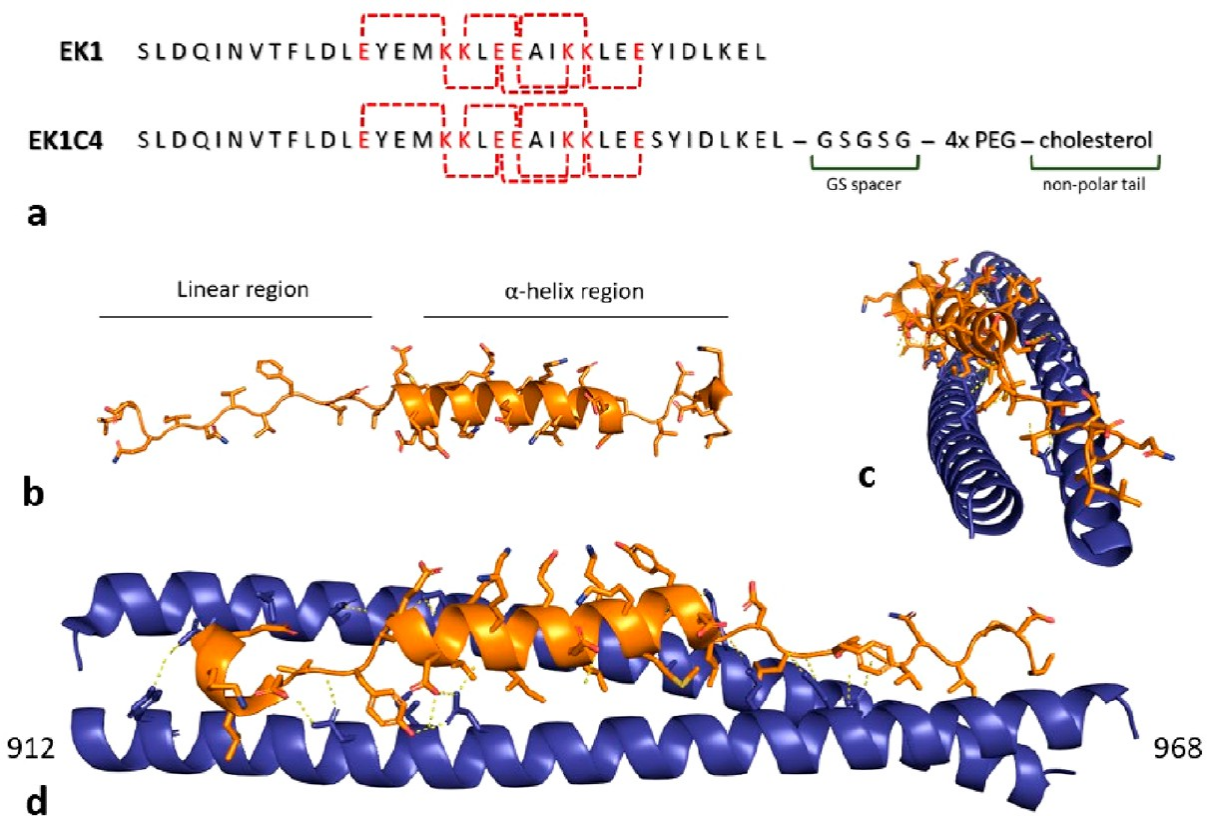
(a) EK1 and EK1C4 structures. Letters highlighted in red represent interhelix salt bridges between charged amino acids. IC_50_ for SARS-CoV-2 PsV infection is 2.4 μM for EK1 and 15.8 nM for EK1C4. (b) Simulation of EK1 folding from its linear amino acid sequence in physiological conditions obtained with PEPFold. (c, d) Crystal of S protein in complex with EK1 peptide (PDB ID 7C53). Blue ribbons represent SARS-CoV-2 S protein HR domain; orange ribbons represent EK1 peptide. Bridge salts at <3.5 Å are represented by yellow dashes.

The rapid spread of SARS-CoV-2 led scientist to develop a pan-coronavirus peptide-like S protein inhibitor, based on the information derived from the previous 15 years of research. The EK peptide library was developed decorating OC43-HR2P, an HCoV-OC43 HR1 fusion inhibitor, by adding K and E residues to gain more salt bridges. In fact, EK1 carries E and K modifications (Figure 11) to increase solubility along with other amino acid mutations away from the HR1 fusion core sequence. EK1 peptide (MW 4.3 kDa, 36 aa) showed the best solubility profile and good anti HCoV-OC43, HCoV229E and MERS-CoV activities *in vivo*, confirmed by prophylactic activity when administered to mice 30 min before virus exposition.^108^ The EK1 peptide organizes in a five-turn α-helix region, which interacts with SARS-CoV-1 and SARS-CoV-2 fusion cores (6HB domain), and in a shorter, linear, region at the C-terminus (Figure 10), in which polar residues interact with a HR1 charged pocket.

EK1 (Figure 11) was refined against SARS-CoV-2. SARS-CoV-1 and SARS-CoV-2 differ by a few amino acid residues in the fusion core region, so as to not affect EK1 binding activity to HR1 complementary coils (Figure 10i,j), but structural enhancements gave better affinity results. Considering that 25-hydroxycholesterol (25-HCh) plays a central role both in viral steroid synthesis and in guest adaptive immunity,^110^ the strategy of protein lipidation with cholesterol-like moieties was explored, after HIV-1/2 LP series inhibition peptides were designed.^109^ 25-HCh can inhibit the infection of various human and animal viruses, including vesicular stomatitis virus, as well as SARS-CoV-2 itself.^109,111^ A library of peptides in which cholesterol or palmitic acid was covalently linked to the C-terminus of EK1, either directly or with a spacer sequence (GSG or GSGSG) and/or a poly-PEG linker between them, was synthesized.^112^ EK1C4 (Figure 11) exhibited the lowest IC_50_ against S-mediated SARS-CoV-2 cell–cell fusion, 1.2 nM, and SARS-CoV-2 PsV infection, 0.8 nM. EK1C4 potently inhibits SARS-CoV-2 replication, with EC_50_ = 36.5 nM, resulting 67-fold more potent than EK1 (EC_50_ = 2.47 μM) in the same assay. Also, the peptide administered to mice by intranasal suspension (0.5 mg/kg) was enough to gain a 100% survival rate if virus exposition occurred within 4 h after the peptide, in comparison to the 20 mg/kg needed for nonlipoylated EK1.

The structure–activity relationship suggests that cholesterol improves pharmacokinetics parameters, and may also be involved in anchoring one of the HR1 trimer grooves, and that the spacer optimal length is 4 units of PEG monomer. The spacer and the linker are long enough to connect the two active moieties.^113,114^

In 2021, Kandeel et al. discovered that, in contrast to SARS-CoV-1, SARS-CoV-2 S-mediated cell–cell fusion cannot be inhibited with HR2 mimicking a minimal length 24-mer peptide.^115^ A 36-mer peptide library including the central helix and residues on the extended N-terminal region sequence were synthesized with one, two, or three mutations according to the improvement of free energy of binding, as described by Dehouck et al. in 2013^116^ (core sequence DISGINASVXNIQKEIDRLXEVAKNLXESLIXLQEL), corroborating the evidence that a higher α-helicity of fusion peptides leads to a higher antiviral efficacy.^117^ Also, as SARS-CoV-2 6HB is a highly conserved region poorly prone to viral mutation, targeting its domain represents an efficient strategy for viral entry blockage, despite the intrinsic instability and short half-life of peptides.

### Pan-Coronavirus Entry Inhibitors: HR Mimicking Peptides against SARS-CoV-2 Variants

Previously synthesized peptides were also screened and optimized against the new SARS-CoV-2 most widespread variants. In 2022, Yang et al.^118^ modeled and synthesized two N-terminally extended HR2 peptides, namely longHR2_42 and longHR2_45, which resulted to be 100-fold more potent than previous pan-coronavirus lipoylated peptide inhibitors.^108,119^ The peptides were conceived so that their N-terminal added extension of HR2 (namely, [KNHTSP]DVDLG-) could interact with HR1 in the HR1–HR2 bundle of postfusion protein, by assuming an extended conformation, with a view to promoting hydrophobic interactions with V1164 and L1166 residues. The treatment with long HR2_42/45 of Caco2-hACE2 cells infected by α and Delta (δ) variants resulted to be effective in the nanomolar range. On the contrary, the IC_50_ against SARS-CoV-2 Omicron increased up to 5-fold, probably due to three further mutations (Q954H, N969K, and L981F), which are directly involved in the interaction with HR1–HR2 bundle structures.^119^

### SL Strategy Applied to Viral Entry Inhibitors

Since the outbreak of the COVID-19 pandemic, the evolution of the virus genotype has been a main drug discovery focus due to the risk of emerging mutations, which could lead to more infectious and lethal variants and, above all, vaccination resistance. The many available crystal structures of the S protein accessible from open access databases such as the PDB combined with NGS of the novel viral strain provide a complete snapshot of the amino acid mutations.^120,121^ Dearlove et al. found more than 7500 polymorphic sites over 17 000 genomes analyzed, corresponding to over 8% of amino acid protein coverage.^122^ Every new main virus variant develops the ability to bind more strongly to the hACE2 receptor. As a matter of fact, the Omicron variant accounts for 30 mutations of the S protein with respect to the wild-type strain, 15 of which localized in the RBD region, and affecting the overall surface potential of the recognition site and providing a tighter binding than the wild type (11.6 vs 22.6 nM).^123,124^ This is caused by the high levels of genetic polymorphisms, such as SNP variation, transcriptional modifications, and post-transcriptional modifications that characterize the S proteins of all the RNA-based viruses.^125^

The most relevant mutations on the RBD are H655Y and N679K common to the α, Mu (μ), and γ variants and associated with increased cell invasion rates, along with increased indirect RBD interactions and enhanced S glycoprotein fusion efficiency.^126−128^

This general viral feature hampers the development of RBD-directed inhibitors against all the main viral variants able to maintain their binding kinetics despite genetic variability, and this is why no ACE2-mimetic peptide has entered the preclinical phase despite their stability and high selectiveness toward their targets in preliminary investigations.^129^ Spike sequence variability determined the therapeutic failure of several antibodies designed against the wild-type and past SARS-CoV-2 viral strains, such as Sotrovimab (VIR-7831) and DXP-604, which failed to recognize the inhibitors due to mutations at the level of K417, L452, E484, and other solvent-exposed residue.^130,131^ On the other hand, although mutations affecting the HR regions are quite common, they do not seem to affect the activity of fusion-mimicking peptides. Indeed, the most frequently mutated positions localize in the noncoaxial helical helices composing the HR1 domain and affect the polar amino acids pointing toward the solvent. Those residues, which tend to be mutated into aromatic amino acids, thus enhancing the entry process thanks to their nonpolar moieties without altering their affinity for fusion peptides, indicate HR regions as favorable druggable sites.^132^

Overall, some of the amino acid substitutions in the Omicron variant have already been associated with clinical outcomes. Mutations Q498R, N501Y, and Y505H seem to increase the virus infectivity by enhancing the binding free energy^121^ and are considered the main determinants targeted by regdanvimab, bamlanivimab, and the Eli Lilly mAb cocktail.^133,134^

**Figure 12:**
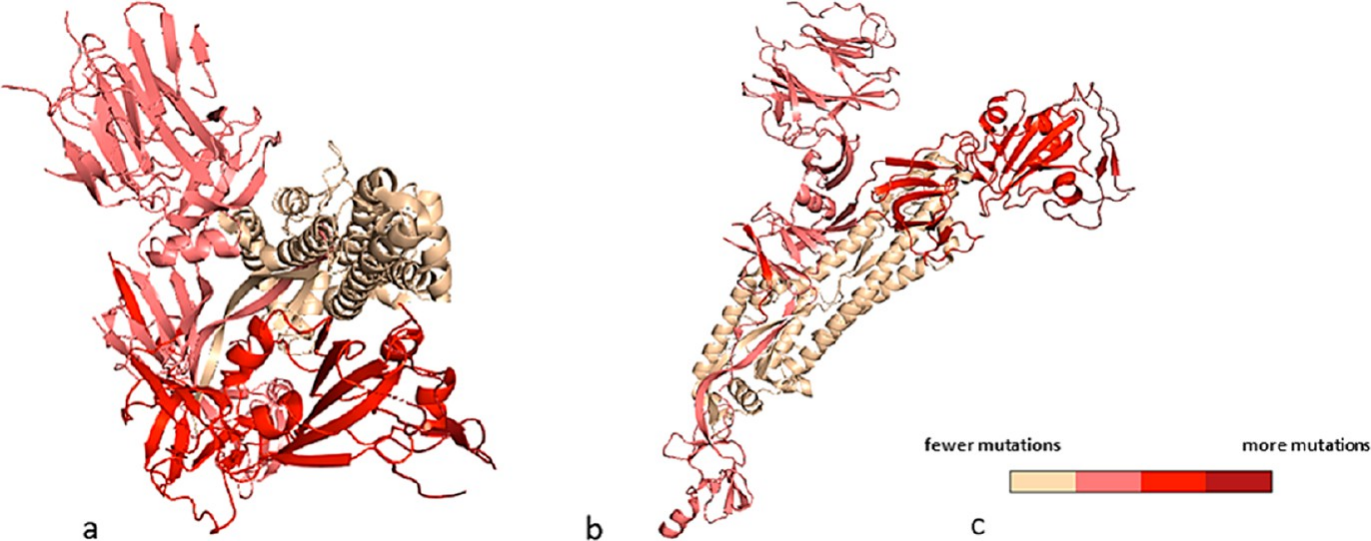
Color code map obtained according to the variability measured by the authorities and reported by the WHO (Tracking SARS-CoV-2 variants (who.int) and ref (136)). Spike protein (PDB ID 6VYB(144), SARS-CoV-2 spike ectodomain structure, Omicron variant, truncated before the TM region): (a) top and (b) side views. (c) Legend. The color key indicates the relative frequencies of single amino acid variations (insertions, deletions, and mutations) for each residue according to the screened single amino acid variants of concerns, from the sequences downloaded from the Global Initiative on Sharing All Influenza Data (GISAID).^136^ The degree of mutation was obtained by dividing the measured frequency incidence by the length of the considered sequence. The quantitative color code map was generated with PyMol (Version 2.5.2). Overall, the lowest mutation rate is associated with the less exposed regions on S2 subunit, whereas, not surprisingly, RBD accounts for the highest mutation frequency.

The rich mutational landscape of spike (see Figure 12) discourages the use of invariant positions to determine a new target that does not allow therapeutic escape. The search for intragenic SLs becomes essential, and the genetic study of the various spike variants will enable us to define them (see Perspective and Foreseen Studies: Alliance between Drug Discovery and Genetics). The SL pairs should be numerous because, after ACE2 recognition and RBD attachment, a massive conformational rearrangement is triggered in the FP along with a repositioning of the HR1 and HR2 domains. These domains arrange in two different conformations, imposing a very high selection pressure on this region and, ultimately, on the residues that compose it. The conformation of the protein prior to ACE2 binding and that of the ACE2-bound spike are achieved through two distinctive and complex amino acid interaction networks. This also imposes a double selection pressure on the residues involved. Thus, even if this region is very variable,^122−124^ an equilibrium between this mutational richness and this double selection pressure cannot be overcome without loss of viral replicability. In other words, whatever the mutations acquired, the virus will always have to preserve both its fitness and its various functions. Thus, therapeutic escape requiring the acquisition of mutations while retaining the protein function is not without cost for the virus, which in most cases loses fitness. Thus, mutations can be achieved and propagated if viruses undergo a first round of mutations stabilizing the function and still retain a degree of fitness, followed by a new set of mutations improving fitness without losing the function (these are the primary and secondary mutations described for HIV). This explains why complex combinations of mutations are preferentially selected. Furthermore, as invariance is achieved at multiple positions within the protein, this indicates the way toward intragenic SLs (see Intragenic Synthetic Lethality).

A hierarchical organization of residue moieties within the HR region is required for a proper folding before and after viral fusion (Figure 10h). This was successfully targeted with the strategy of mimicking peptides and small chemical compounds, and/or peptides can be designed to disrupt the HR organization. Therefore, an SL medicinal chemistry strategy requires the design of inhibitors with moieties which induce the variation of single amino acids of the fusion domain. This is possible by altering the hindrance of critical residues essential for fusion core formation, causing the virus to select viral mutants carrying amino acid substitution able to escape the inhibitor but unfavorable to assemble properly the HR core. However, due to the overall low druggability of the S protein itself, and its lack of binding pockets, we suggest that SL strategy should be more easily applied to other viral proteins, especially its enzymes (e.g., soluble hydrolases and polymerases).

So far, the antigenic comparison technique is preferably carried out by immunobinding assays. However, the high mutation rate of the S protein and the vaccine industry manufacturing prompted a strong focus on learning platforms to predict the evolutionary epitope variations.^135^ The SARS-CoV-2 S glycoprotein is represented in Figure 12 as a heat map, indicating the more variable residues in the Lambda and Omicron variants compared to the wild-type virus. Here one can visualize immediately how the RBD, represented in dark red, carries half the mutations (15) of the whole S protein in between the wild-type and Omicron viruses. The heptad repeat (HR) region is the highest conserved.

### Entry Inhibitors Targeting the Transmembrane Serine Protease, Type 2 (TMPRSS2)

One of the two entry-fusion paths of β-coronaviruses, including SARS-CoV-2, is represented by the mutual recognition of spike and ACE2 of TMPRSS2 (transmembrane serine protease, type 2) mediated on the DPSKPSKR↓SFIED sequence. Such an entry pathway does not involve endosomal uptake but allows the virion to fuse its envelope to the host cell membrane and to release its genic and proteinaceous material into the cytosol.^137^ This infection strategy has been named “early pathway” at the plasma membrane fusion.^138^ TMPRSS2 (492 aa) upregulated expression is associated with epithelial cells of several tissues, including gastrointestinal, respiratory, and urinary systems.^139−142^

While MERS-CoV needs TMPRSS2 to cleave away S1 from the S2 subunit at the furin domain, SARS-CoVs do not strictly need a such prefusion activation, although SARS-CoV-2 still contains a furin-like cleavage site between subunits S1 and S2 (PRRA multibasic site). This can be employed by the virion polyprotein convertase and enhances human viral pathogenicity.^143−145^ For these reasons, selective protease inhibitors against TMPRSS2 have been studied since the SARS-CoV-1 outbreak.

Camostat mesylate, or FOY-305 (Table 3A), a trypsin-like serine protease inhibitor, initially developed to treat chronic fibrosis and approved in Japan for the treatment of chronic pancreatitis,^146^ also showed a good tropism for the airway epithelial sodium channel (ENaC),^147^ making it an ideal candidate for SARS-CoV investigation.

**Table 3:**
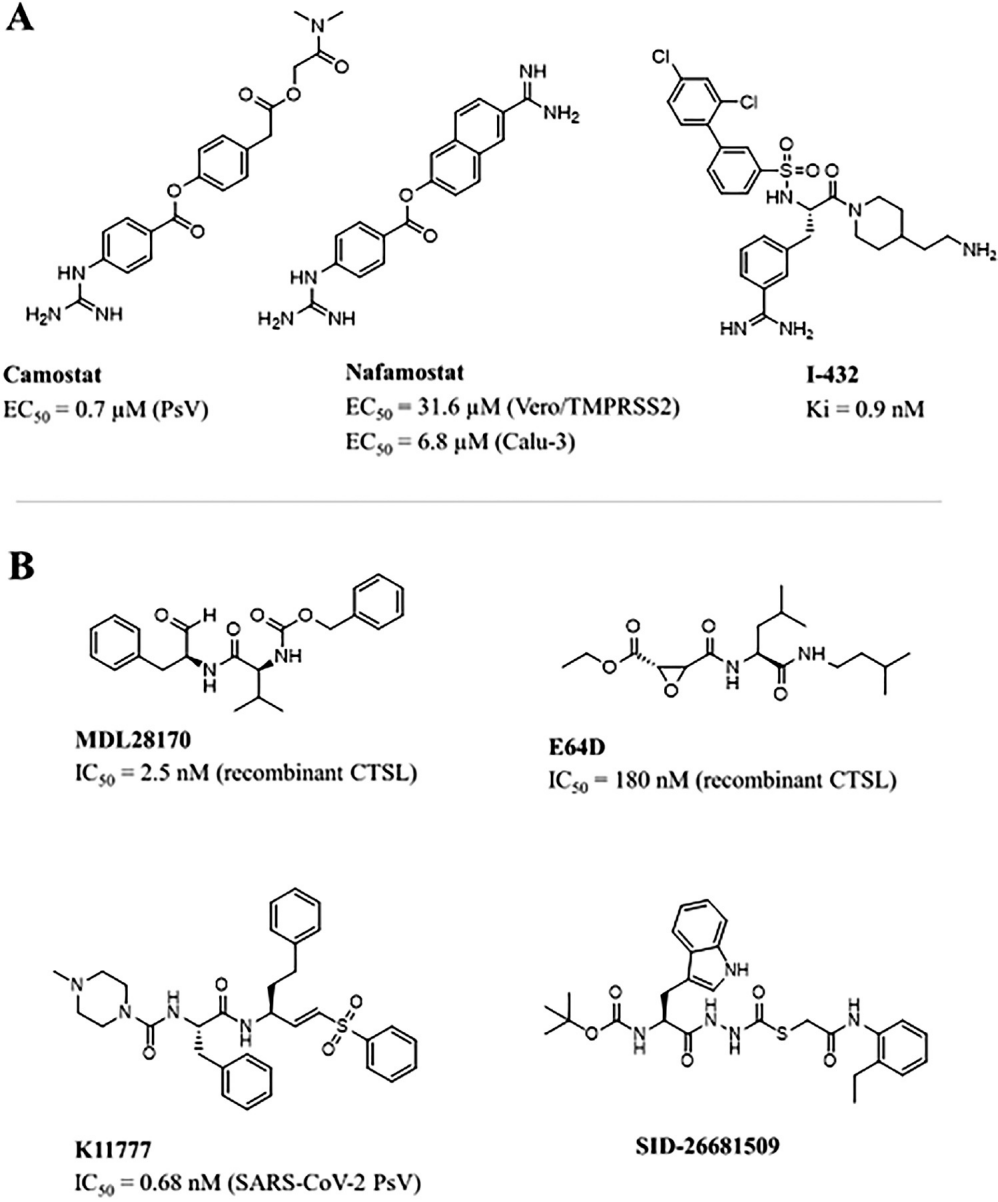
Chemical Structures of Main TMPRSS2 Inhibitors (A) and Cathepsin L Inhibitors (B)

**Figure 13:**
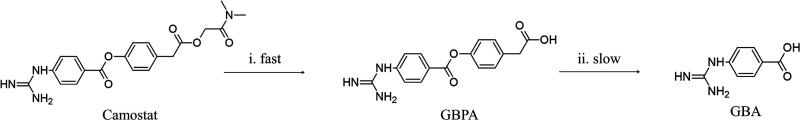
Metabolism of camostat when administered to transformed HEK-293T cells. Its derivative GBPA (4-(4-guanidinobenzoyloxy)phenylacetic acid) still inhibits TMPRSS2 (although with a 100-fold reduction), while GBA (4-guanidinobenzoic acid) is inactive.

**Figure 14:**
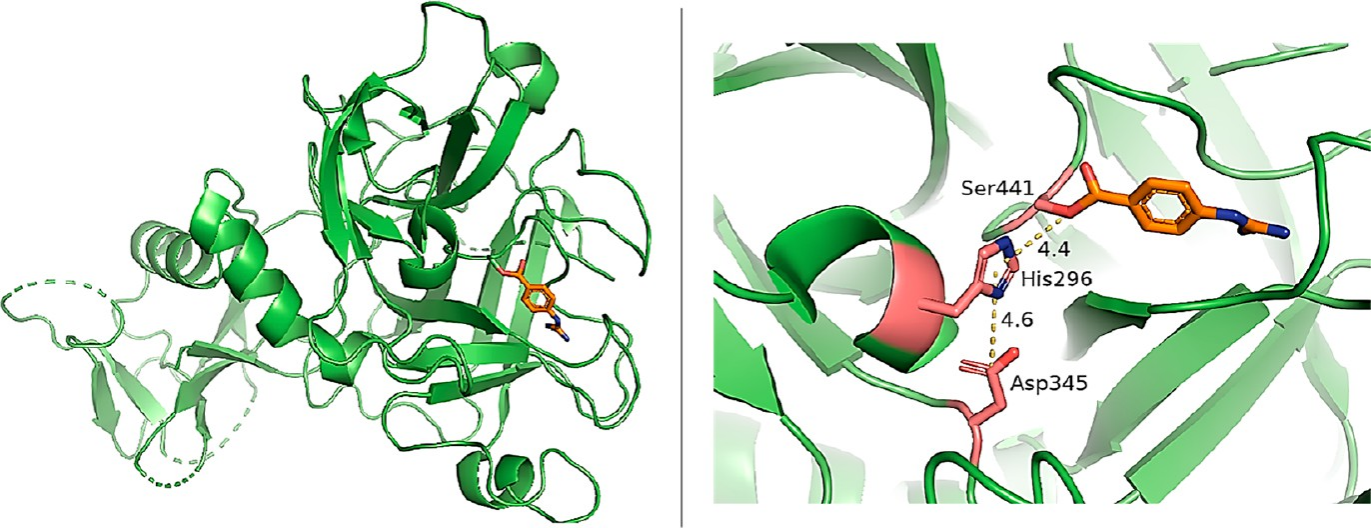
(left) TMPRSS2 in complex with the camostat/nafamostat fragment GBA (PDB ID 7MEQ(151)) and (right) a focus of its binding site in the S1 hydrolytic pocket of the enzyme. The catalytic triad of the enzyme (Ser441, His296, and Asp345) cleaves the inhibitor at its ester function, which results in the formation of a covalent bond between Ser441 and the carbonyl group of the remaining GBA.

Although the proper molecular mechanism of action for camostat is still under investigation, it seems to act as a prodrug. After the hydrolysis of the *N*,*N*′-dimethylamminoethylic moiety to the active metabolite 4-(4-guanidinobenzoyloxy)phenylacetic acid (Figure 13), a benzyl ester group is uncovered to be reversibly acylated by a serine residue of the inhibited protease.^148^ From the crystal structure of camostat complexed with the urokinase-type plasminogen activator (uPA), Sun et al.^149^ identified the hydrolyzed portion GBPA covalently bound to a serine residue, resulting in an IC_50_ for TMPRSS2 between 4 and 6 nM. In particular, the guanidine group points toward the bottom of the S1 pocket, into the catalytic triad (Asp345, His296, and Ser441).^149^ A valine-rich lipid pocket accommodates the dimethyl amide tail, and the guanidine moiety hydrogen binds to Asp435. The active mechanism proposed through MD simulation involves Ser441 deprotonation of TMPRSS2 His296, which acts as a nucleophile toward the camostat ester function, with subsequent nucleophilic acylation and the cleavage of the hydrophobic tail. When the complex between the cleaved camostat and TMPRSS2 is formed (Figure 14), the enzyme active site is modified and the protease activity is blocked.^150^

TMPRSS2 specific inhibition was already observed in MERS-CoV *in vitro* models, where it has been proven that Vero-TMPRSS2 infected cells were protected by camostat, unlike Vero E6 cells, along with a 270-fold reduction of MERS-CoV production in Calu-3 cells.^152^*In vivo* inhibition assays on mutated SARS-susceptible mice, which were administered camostat mesylate for 9 days prior to viral exposition, registered an ∼60% survival rate to SARS-CoV-1. This suggests SARS-CoV-1 depends on serine protease activity for viral spread *in vivo*,^153^ although higher doses of camostat mesylate (30–50 mg/kg) did not reduce lethality. Camostat mesylate is currently in a phase II double-blind clinical trial for COVID-19 syndrome involving 596 participants at the Hôpitaux de Paris (France) with a dose of 600 mg/day *per os* (EudraCT No. 2020-003366-39) and at Yale University with a dose of 200 mg, 3 times daily, for 7 days (ClinicalTrials.gov: NCT04353284).

Nafamostat mesylate (Table 3A), studied as an immediate acting anticoagulant,^154^ displays shares the same molecular mechanism of action as camostat, but has a higher logP and a lipophilic tail. With a higher affinity for TMPRSS2 than camostat, MERS-CoV inhibition properties of nafamostat were studied on *in vitro* fusion assay, and an IC_50_ of ∼1 nM on Calu-3 infected cells was registered.^155^ Furthermore, nafamostat blocks SARS-CoV-2 entry into host cells with roughly 15-fold higher efficiency, with a 50% IC_50_ in the low-nanomolar range,^156^ probably due to the formation of a higher number of hydrogen bonds (five) with the substrate.^157^ When nafamostat mesylate is administered to Vero E6/TMPRSS2 recombinant lines infected with SARS-CoV-2, its displays an EC_50_ of 31.6 μM. The ongoing MD studies on these molecules suggest that improved inhibitors should contain hydrogen bond donors and positively charged moieties that could interact principally with Asp435, together with a more hydrophobic moiety to accommodate a valine-rich loop. Nafamostat is programmed to undergo a prospective clinical study (RACONA Study) for the treatment of COVID-19 at Padua University Hospital (Italy), in collaboration with Yokohama University and Yokohama City University (Japan) and University of Zurich (Switzerland). The studies are still in the recruitment phase (ClinicalTrials.gov: NCT04352400). With the recent spreading of the new viral variants, several authors have highlighted the atypical entry path of the Omicron virus, which presents few mutations in the furin cleavage site (e.g., T716I and N679K), thus seeming to prefer the endocytic entry to the S1/S2 cleavage.^158^ As *in vitro* models showed Omicron to be more infectious even in the presence of TMPRSS2 inhibitors and the large number of cationic residues favors the recruitment of endocytic hydrolases like cathepsins involved in the endocytic pathway, this seems to be the most likely entry mechanism.^159^

### Arylsulfonylated Amides

Another study on TMPRSS2, although not related to SARS-CoV-2, showed *in vitro* catalytic activity on porcine small intestinal epithelial (IPEC-J2) cells and suggested that structures containing a 4-amidinobenzylamide as residue, and several arylsulfonylated amides of 3-amidinophenylalanine, could act as nanomolar TMPRSS2 inhibitors, too.^160^ Derivatives of 4-amidinobenzylamide containing a small, polar amino acid like moiety substituting the sulfonamide nitrogen are well-known inhibitors of HAT, matriptase, and thrombin, but they also gain a TMPRSS2 inhibitory action when a 4-substituted piperazine or piperidine derivative side chain is inserted.^161^ I-432 (Table 3A), with a *K*_i_ of 0.9 nM for TMPRSS2, was screened *in vitro* using a fluorescence-based method to define enzymatic inhibition at different concentrations.^161^ Although β-coronaviruses were not the intended targets for this molecule, TMPRSS2 inhibition properties should be investigated in the search for compounds capable of preventing viral spread.^162^ It is worth noting that TMPRSS2 also proved to be involved in proteolytic activation of some influenza A viruses, promoting their infectivity,^163^ and that the HA10 subtype of the avian influenza A virus (AIV) was unable to infect the TMPRSS2 knockout mice, since the enzyme is indispensable for the proteolytic cleavage of the viral hemagglutinin (HA). Although only preliminary results have been obtained with SARS-CoV-2, human TMPRSS2 genotypic variants (already present in some cancer tissues) further enrich the knowledge of the role of this protease on viral entry.^164,165^

### Entry Inhibitors Targeting Cathepsin L

As the endosomal pathway requires the intervention of cathepsin L (CTSL) for endolysosomal escape, the inhibition of this cysteine exopeptidase implicated in cell growth, matrix regulation, and neovascularization represents a target for the development of antivirals targeting β-coronaviruses and other viruses such as the Ebola one.^99^ Cathepsin L inhibition *in vivo* treatment decreased entry of SARS-CoV-1 and SARS-CoV-2 pseudovirions into 293/hACE2 by over 76%, suggesting cathepsin L, but not cathepsin B nor calpain, is responsible for an endosomal S1/S2 spike cleavage.^166^ MDL28170 (or calpain inhibitor III, or Z-Val-Phe-CHO) (Table 3B), was identified as an efficient inhibitor of CTSL-mediated substrate cleavage, with an IC_50_ of 2.5 nM in a high-throughput screening, together with good inhibition toward SARS-CoV-1 infection.^167^ Calpain inhibitors were classified as candidates against SARS-CoV-1, and MDL28170 was assayed along with analogous di- or tripeptide aldehydes like Z-Leu-Nle-CHO and Z-Leu-Leu-Tyr-CH2F. MDL28170 displayed the best result in a neutral red uptake assay, with an EC_50_ of 0.5 μM in African green monkey kidney cells (Vero 76).^168^ Docking results suggest that two hydrogen bonds are involved in CTSL inhibition: one between the carbonyl oxygen of MDL28170 and the side chain nitrogen of His163 of CTSL and another between Cys25 catalytic thiol side chain residue and the other carbonyl oxygen of ligand, while the end term aromatic ring is accommodated in a large hydrophobic pocket of the enzyme.^169^ Unfortunately, when tested against SARS-CoV-1/2, no direct antiviral effect emerged from viral titration studies. However, this compound can be used as a driving core for further drug development.^168^ The compound E64D (Table 3B), an irreversible, covalent, membrane-permeable inhibitor of lysosomal and cytosolic cysteine proteases and specific against cathepsin L, was initially tested *in vitro* on SARS-CoV-1 and MERS-CoV and showed a reduction in the entry of of SARS-CoV-2 PsV by 92.5%. Its structure contains an epoxide ring as a warhead that can undergo nucleophilic attack by the thiol of the catalytic Cys residue to form with it a covalent bond. Due to this nonspecific mechanism of action, it could target both catepsins L and B. However, as its structure occupies the prime site at the enzyme pocket of CTSL, this improves its specificity.^170,171^

Teicoplanin, a semisynthetic glycopeptide antibiotic with a broad spectrum of activity, exhibits good activity against SARS-CoV-2 pseudovirion entry with an IC_50_ of 1.6 μM,^172^ whereas the chemical probe and cysteine protease inhibitor K11777 (Table 3B) is able to inhibit pseudovirus entry with an IC_50_ value of 0.68 nM.^153,172^ The sulfonyl vinyl moiety of the molecule acts as a Michael acceptor and reacts with Cys-SH in the catalytic pocket of cathepsin L/B, and structure–activity studies identify phenetyl groups as selectivity drivers toward cathepsin L, while 2-naphthyl groups are more specific to cathepsin B, as CTSB only tolerates aromatic residues in its lipophilic pocket.^173^ The hydrazide-derived peptidomimetic SID-26681509 (Table 3B) was demonstrated to decrease SARS-CoV-2 pseudovirion entry by about 76% at 2 μM.^170^

### Entry Inhibitors Targeting PIKFYVE and Protein Kinase B (Akt)

PIKFYVE is a ubiquitous, highly conserved lipid kinase, responsible for the phosphorylation of phosphatidylinositol-3-phosphate (PI_3_P) to phhosphatidylinositol-3,5-diphosphate (PI_3,5_P), involved in lysosome fusion to endosomes and vacuolation trafficking.^174^ Apilimod (Table 4), a small molecule that inhibits the phosphotransferase activity of PIKFYVE with an IC_50_ of 14 nM and developed as a suppressor of IL-12 and IL-23 production, was first tested against Ebola.^175^ The compound was shown to prevent EVD (Ebola virus disease) infection of several cell lines in the range of 0.1 μM concentration, with a dose-dependent response.^176^ The general mechanism of action suggests that apilimod activity on PIKFYVE results in a late endolysosome inhibited microtubule mediated vesicular trafficking.^177^ MERS-CoV *in vivo* models also show that NAADP Ca^2+^ channels on endosomal membranes are under expressed when the PIKFYVE pathway is inhibited, resulting in poor vesicular organization.^178^ As apilimod showed good properties against murine hepatitis virus (MHV), SARS-CoV-1, and MERS-CoV PsVs, it was tested on 293/hACE2 cells exposed to SARS-CoV-2, but the low blood availability made it difficult to test its activity *in vivo*.

**Table 4:**
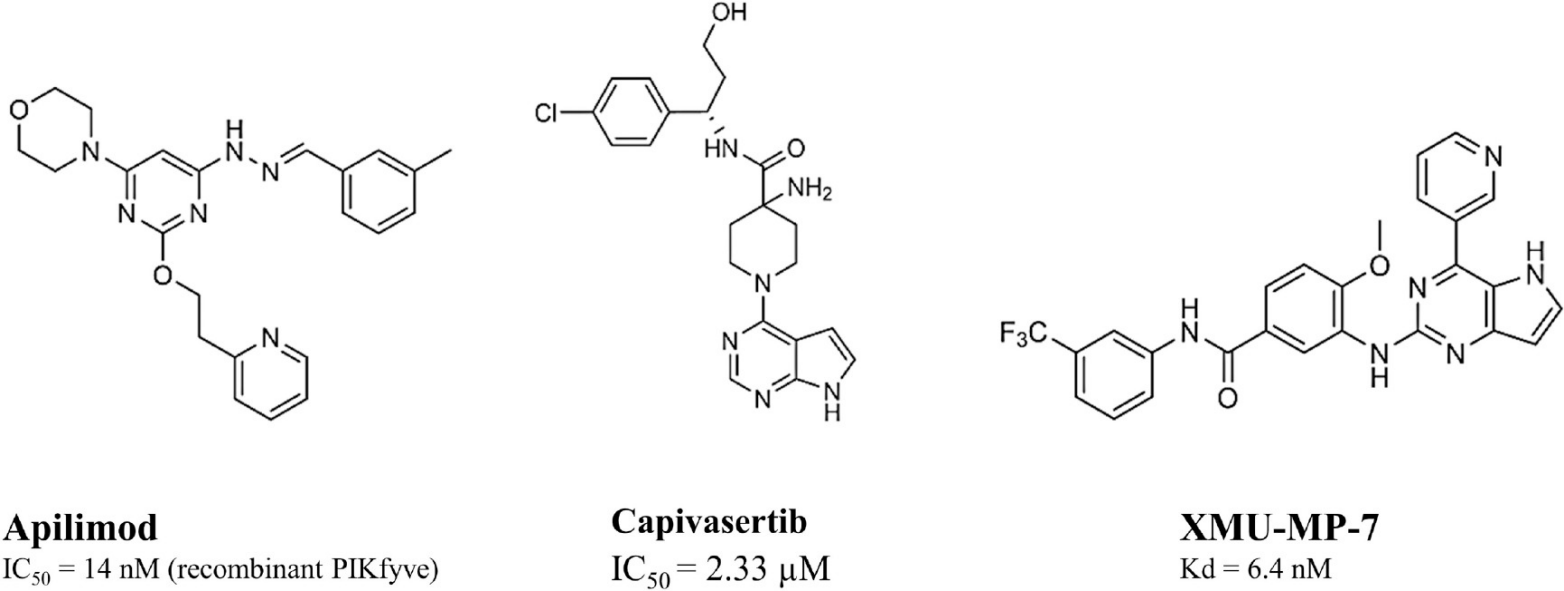
Examples of PIKFYVE and Akt Inhibitors

Protein kinase B (also known as Akt) was considered as a putative target for SARS-CoV-2 fusion inhibitor, as part of the most important kinase signaling of SARS-CoV-1 infection. *In vivo* luciferase activity on the PsV tested an IC_50_ of 2.33 μM for the anticancer drug capivasertib (Table 4), with a 50% cytotoxicity concentration on Vero cells of 82 μM.^179,180^ The low toxicity of this compound makes it a good lead for further investigation for managing COVID-19 positive patients in comorbidity with neoplastic formations. Recently, a series of 2,4-disubstituted-5*H*-pyrrolo[3,2-*d*]pyrimidine derivatives were demonstrated to have higher Akt inhibitory activity compared to apilimod.^181^ Among them, XMU-MP-7 (Table 4) achieved satisfying viral inhibition at 200 nM, demonstrated good *in vivo* pharmacokinetics, and resulted in low EC_50_’s against the most common SARS-CoV-2 variants (EC_50_’s of 12.4 nM against β variant and below 6.9 nM against γ, δ, and o variants).

Overall, since PIKFYVE and Akt inhibitors maintain their antiviral activity across the entry and postentry stages of SARS-CoV-2 infection, they represent highly promising leads in antiviral drug discovery.^181^ However, these compounds have recently been reported to be poorly active in suppressing COVID-19 associated inflammation in murine models, suggesting the need for further optimization.

## RNA-Dependent RNA Polymerase

**Figure 15:**
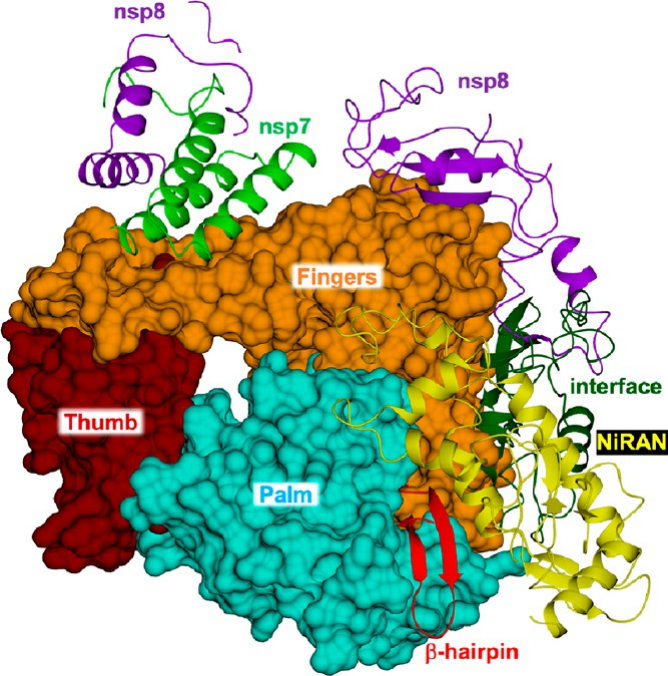
Structure of the RdRp subunits nsp12, nsp7, and nsp8 of SARS-CoV-2 (PDB ID 6M71(189)). In nsp12, the polymerase subdomains are colored as follows: NiRAN (yellow cartoon), β-hairpin (red cartoon), interface (dark green cartoon), fingers (orange surface), palm (cyan surface), and thumb (tan surface). Nsp7 and nsp8 are displayed as green and purple cartoons.

SARS-CoV-2, as many positive-stranded RNA viruses, is characterized by a complex replication/transcription process governed by many factors belonging both to the host cells and to the virus. The replication process is completed and controlled in the cytoplasm of the host cell by an RNA-dependent RNA polymerase complex. This multivalent enzyme involves different subassembly units of nonstructural proteins (nsp’s), encoded in the ORF1ab region of the virus genome open reading frame.^33,182^ A key component of this complex scaffolding is the RNA-dependent RNA polymerase (nsp12).^183,184^ This enzyme alone possesses minimal activity and requires association with the cofactors nsp7 and nsp8 to improve template binding and processivity.^185^ Thus, the nsp12–nsp7–nsp8 complex (Figure 15) represents the catalytic machinery for nucleotide polymerization.^185,186^ To date, no structural information is available on the isolated nsp12, whose structure has been reported only in complex with its cofactors, limiting the comprehension of its peculiar activation mechanism. The RdRp function is pivotal among coronaviruses, its primary structure being highly conserved in SARS and MERS CoVs.^187^ RdRp proteins of SARS-CoV-1 and -2 share 96% amino acid identity with a high degree of similarity in the nucleoside binding domain, and the antiviral drugs already developed to treat SARS-CoV-1 and MERS-CoV infections represent a resource to quickly identify effective drugs against SARS-CoV-2. To date, structural information on this target is available only for SARS-CoV-1 and -2. Nsp12 consists of three domains: the nidovirus-unique N-terminal extension, the interface, and the C-terminal RdRp domains. The role of the nidovirus-unique N-terminal extension domain, adopting a nidovirus RdRp-associated nucleotidyl transferase (NiRAN) architecture, remains to be fully elucidated. On the other hand, the C-terminal RdRp domain is involved in template binding, nucleoside triphosphate (NTP) entry, and polymerization. This domain resembles a cupped right hand and comprises the fingers, palm, and thumb subdomains (Figure 15). Minor differences have been reported between the nsp12–nsp7–nsp8 complexes of SARS-CoV-1 and -2. In SARS-CoV-2 the N-terminal β-hairpin is in the groove clamped by the NiRAN and the RdRp palm subdomain, where it stabilizes the overall structure through a set of close interactions (Figure 15).^182,184,188,189^

The catalytic site of RdRp is characterized by seven conserved polymerase motifs, named A–G. Motifs A–E are in the palm subdomain, whereas motifs G and F are part of the fingers’ subdomain. Motif A contains two binding sites for bivalent metal ions, attributed to zinc ions and likely serving as structural components to maintain the integrity of the RdRp architecture.^184,188^ Two aspartate residues, Asp760 and Asp761 belonging to motif C, are essential for the RNA synthesis. In RdRp, few channels are found to connect the catalytic site to the exterior, lined by positively charged residues that facilitate the entry of NTPs and the template strand, on one side, and the exit of the nascent strand on the other. The RdRp complexes reported so far include a variable number of nsp8 units.^182,184,188,189^ The nsp7–nsp8 heterodimer binds to the nsp12 on the polymerase thumb domain facing the NTP entry channel. Binding in this position sandwiches the index finger loop of nsp12 between the nsp7–nsp8 heterodimer and the polymerase thumb domain. The interaction of the nsp7–nsp8 heterodimer with this loop has been proposed to facilitate the interaction of nsp12 with additional components of the RNA synthesis machinery. A further nsp8 unit binds to the nsp12 interface domain proximal to the fingers’ domain and the RNA template-binding channel. Nsp12 and nsp8 have been proposed to interact with a large set of other nsp’s, suggesting that they form a protein–protein interaction hub within the viral replication complex.^184^ The interfaces among subunits could represent new promising targets for the development of antiviral drugs. A recent structure of the SARS-CoV-2 RdRp (PDB ID 6YYT(182)) has shown that the two conserved N-terminal regions of nsp8 are implicated in the enzyme consecutive reactions without releasing its substrate (processivity). Indeed, in the complex with RNA, these nsp8 extensions fold in α-helices that flank the protruding RNA duplex exiting from the RdRp cleft.^182^ The key role played by the cofactors in the polymerase activity suggests the interfaces among subunits as new promising targets for the development of antiviral drugs.^190,191^ Key contacts of RdRp with other viral proteins, such as nsp7 and nsp8, in replication transcription complexes were pointed out, leading the way to the development of protein–protein interaction inhibitors.^192^ Increasing knowledge of the mutational rate and quality of the mentioned interfaces opens the way for exploring the SL strategy, thus limiting the therapeutic escape. However, more data are needed.

Very recently, Tian et al.^193^ reported a comprehensive state of the art study on the RdRp inhibitors. Many of them were effectively developed and are currently in use to cure several diseases such as hepatitis C, hepatitis B, influenza A, and influenza B, whereas others are in clinical or preclinical phase. RdRp inhibitors can be subdivided in two main classes of compounds, based on their chemical structures and protein-binding domains: nucleoside analogue inhibitors (Nis) and non-nucleoside analogue inhibitors (Nnis). The first class of compounds is responsible for the termination of the RNA chain elongation during the virus replication process, through their incorporation by the RdRp complex active site into the elongating chain, while the second class acts by blocking the protein by binding at allosteric sites.^193−195^

The current need for efficient drugs to cure COVID-19 prompted researchers to consider RdRp inhibitors in view of repurposing strategy. Currently among all nucleoside analogues developed over the years, only a few have obtained approval for managing COVID-19 disease, as in the case of remdesivir, which was approved by FDA on October 22, 2022.^190,196−200^ More recently molnupiravir (EIDD-2801) has been authorized from the medicine regulator of the United Kingdom as the first oral drug to treat adult patients with mild to moderate COVID-19 infections and with risk factors for severe disease. The drug is currently in clinical phase II/III and has been shown to significantly decrease the risk of hospitalization or mortality.^201,202^ The cryo-EM structure of SARS-CoV-2 full-length RdRp nsp12–nsp7–nsp8 at a resolution of 2.9 Å was solved by Gao et al.^188^ followed recently by that of the complex with the active form of remdesivir by Yin et al.^189^ These structures helped to clarify the remdesivir mechanism of action and provide the rational basis for the design and optimization of other parent nucleotide drugs which have shown activity in blocking SARS-CoV-2 in cell-based assays.^203,204^

### Nucleoside Analogue Inhibitors (Nis)

**Figure 16:**
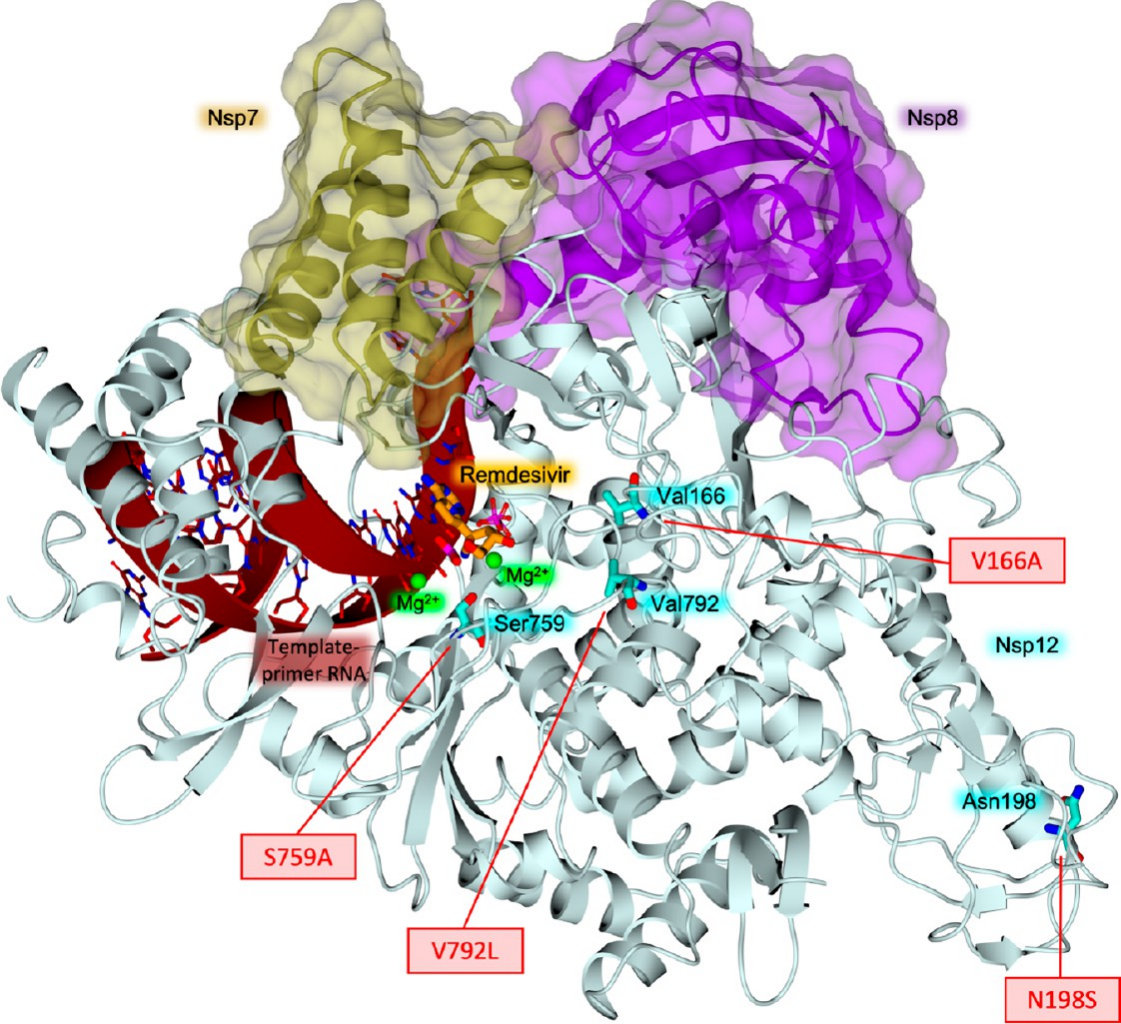
Structure of RdRp (nsp12–nsp7–nsp8 complex) bound to the template-primer RNA and the triphosphate form of remdesivir (PDB ID 7BV2(189)). The identified mutations on nsp12, connected with resistance to GS-441524, are highlighted. Nsp12 is shown in a light cyan cartoon; target residues and remdesivir are in sticks (cyan and orange carbons, respectively). Nsp7 and nsp8 are shown in gold and purple cartoons, respectively, surrounded by their surfaces. The template-primer RNA is shown in dark red, and magnesium ions are displayed as green spheres (arbitrary radius).

**Figure 17:**
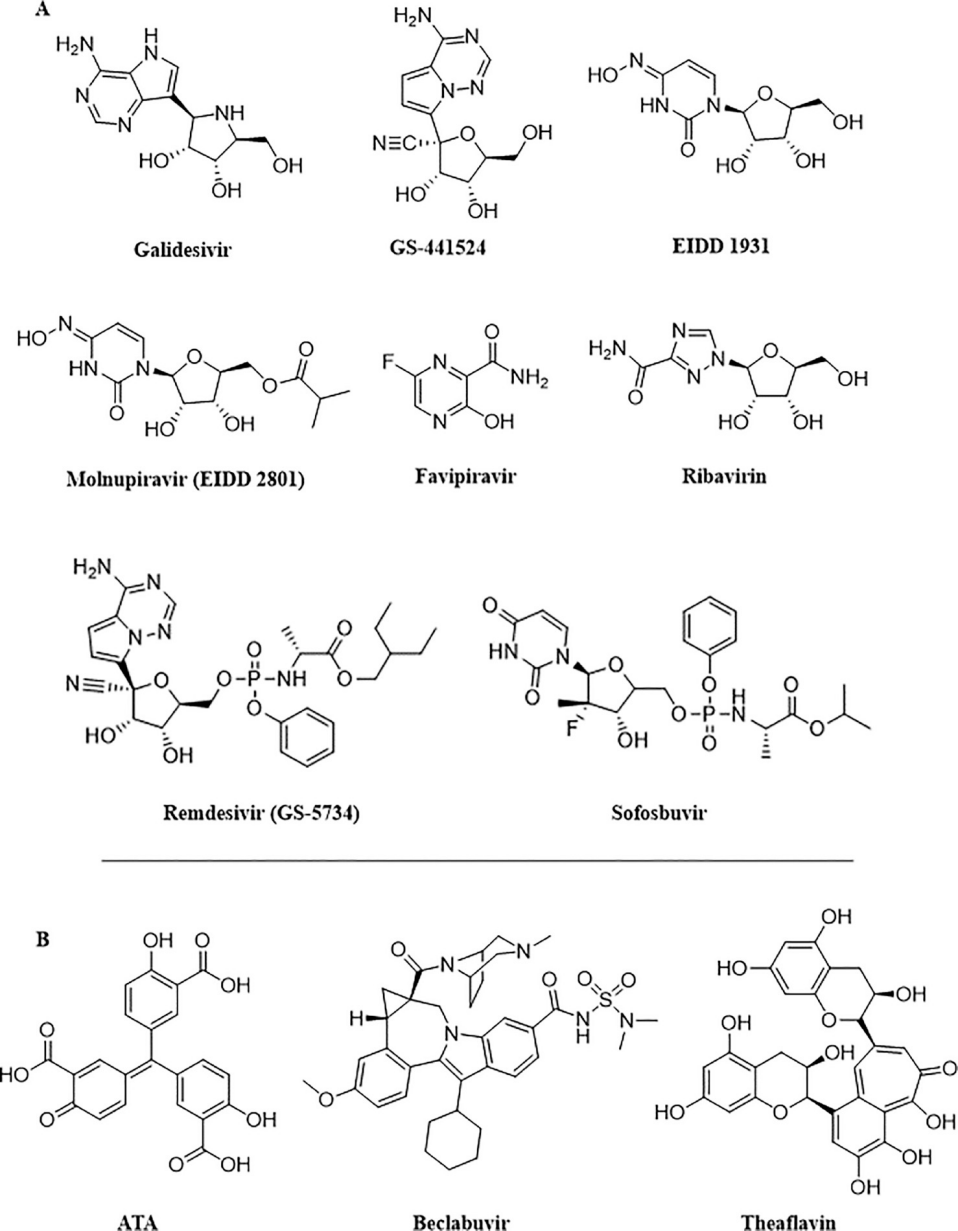
Chemical structures of nucleoside analogue inhibitors (Nis, panel A) and non-nucleoside analogue inhibitors (Nnis, panel B).

Remdesivir (RDV, GS-5734), a monophosphoramidate, is a prodrug of the active compound GS-441524 (Figures 16 and 17A) bearing a free 5′-hydroxy group. It is a broad-spectrum inhibitor of the replication of viral genome in several RNA viruses by interfering with the nsp12 RdRp.^205^ The resolution of the SARS-CoV-2 RdRp in complex with remdesivir shows the enzyme incorporates the drug as triphosphate into the RNA chain. Once entered, the inhibitor blocks RNA synthesis due to the lack of incorporation to its monophosphate moiety of a complementary uridine triphosphate (UDT).^206−208^ In addition, remdesivir shows different interactions between the cyano group and Ser861 present in the exit channel, thus preventing RdRp translocation and delaying chain termination after its incorporation.^196,209,210^ Remdesivir was initially developed to interfere with Ebola virus replication, and further studies demonstrated that Ebola’s RdRp incorporated ATP and remdesivir-TP with the same efficiencies. The selectivity of remdesivir-TP increases up to 500-fold, with respect to ATP alone, when the purified human mitochondrial RNA polymerase (h-mtRNAP) is used.^190^ Recently remdesivir has been proposed as a promising antiviral drug candidate against a wide number of RNA viruses including SARS-CoV-1 and MERS-CoV.^211^ In fact, it inhibits both MERS-CoV (IC_50_ = 0.074 μM) and SARS-CoV-1 (IC_50_ = 0.069 μM) replication in human airway epithelial (HAE) cell models with broad-spectrum activity against various bat coronaviruses.^212^ In Vero E6 cells experiments, remdesivir was able to block SARS-CoV-2 infection at a concentration of 0.77 μM of IC_50_ with low cytotoxicity (CC_50_ > 100 μM).^213^ Gottlieb et al.^214^ reported that treatment of COVID-19 patients with remdesivir reduced hospitalization by 87% compared to placebo.

Although this proved the efficacy of the drug, it has also been reported that an immunocompromised patient developed drug resistance. It is therefore necessary to start investigating and understanding the pathways of resistance to remdesivir.^214^ Stevens et al. recently published a study exploring the potential developmental pathways leading to remdesivir resistance, the molecular mechanisms involved, and the viral determinants.^215^ They demonstrated that SARS-CoV-2 develops resistance to GS-441524 *in vitro*, and in particular, after 13 passages of the drug, the EC_50_ increased 2.7–10.4-fold. Sequence analysis identified mutations in the active RdRp motif S759D, V166A, N198S, S759A, and V792L (Figure 16). Biochemical analysis showed that S759A is responsible for the decreased preference for remdesivir-TP as a substrate by the nsp12 RdRp, whereas nsp12 V792I diminishes the uridine triphosphate concentration needed to overcome template-dependent inhibition associated with RDV (Figure 16).^215^

Molnupiravir (EIDD-2801) is a 5′-isopropylester prodrug of β-d-*N*^4^-hydroxycytidine (EIDD-1931) (Figure 17A). This latter inhibits SARS-CoV-2, MERS-CoV, and SARS-CoV-1 replication with EC_50_ in the nanomolar range. Upon *in vivo* conversion to EIDD-1931 5′-triphosphate, the activated form is incorporated into the RNA chain by RdRp CoV causing nonobligate RNA chain termination. Molnupiravir inhibits the SARS-CoV-2 replication with 5–10-fold higher efficacy than remdesivir. The potency improvement seems to depend on two additional hydrogen bonds established by the drug in the central pocket of RdRp. In particular, the side chain residue Lys545 forms a hydrogen bond with the N(4)-hydroxyl group present in the drug structure and the guanine base in the template strand can establish a hydrogen bond with the cytidine base.^204,216,217^ Recent trials (NCT04575597) showed that molnupiravir, the first oral anti-SARS-CoV-2 drug available, decreases both hospitalization and the risk of death in patient with moderate COVID-19.^195^ Like remdesivir and molnupiravir, other analogues such as favipiravir, ribavirin, and galidesivir (Figure 17A) showed SARS-CoV-2 inhibition at the cellular level, and they have been proposed as potential inhibitors of the viral RdRp through nonobligate RNA chain termination upon conversion to the triphosphate derivative. All these molecules carry the entire ribose group which is responsible for stable hydrogen bonds with the central channel of the protein. Favipiravir undergoes phosphoribosylation *in vivo*, it is recognized by the RdRp enzyme as a purine base, and it is incorporated into the viral RNA causing replication to be interrupted.^218^ Multiple clinical trials including favipiravir alone and in combination with other antiviral drugs are ongoing to investigate and optimize the potentiality of this compound as an anti-SARS-CoV-2 drug. Ribavirin, a synthetic nucleoside analogue inhibitor that showed a preclinical beneficial effect to treat SARS-CoV and MERS-CoV infections when it was administered in combination with IFN-α, requires further clinical investigations to assess previous observed side effects.^219,220^ Galidesivir (BCX4430) is a broad-spectrum antiviral adenosine analogue initially developed to treat hepatitis C virus (HCV) which proved active toward several viruses such as Ebola, Marburg, yellow fever, Zika, and SARS-CoV-2. Based on the replication mechanism similarity between HCV and CoV, and on a structure–activity relationship analysis of HCV inhibitors, Ju et al.^221^ proposed that the FDA-approved hepatitis C drug EPCLUSA (sofosbuvir/velpatasvir) should inhibit coronaviruses, including SARS-CoV-2. Sofosbuvir (Figure 17A), a pyrimidine nucleotide analogue able to inhibit the HCV RdRp NS5B,^222,223^ is converted *in vivo* into the triphosphate (2′-F, Me-UTP) which binds in the active site of the RdRp stopping the RNA chain elongation, thus preventing the viral growth.^224,225^ The repurposing approach proved useful as the drugs already approved have a known toxicity profile and have greatly shortened the development time. Along this line, recently Parvez et al. designed a pharmacophore using remdesivir and, after having undertaken a molecular docking analysis, they were able to select two compounds from the ZINC database, namely ZINC09128258 and ZINC09883305, that effectively bound the RdRp of SARS-CoV-2.^226^

### Non-Nucleoside Analogue Inhibitors (Nnis)

Unlike Nis that bind the catalytic site of RdRp, Nnis bind to allosteric sites, interfering with the functionality of the protein. The active site of the RdRp has been shown to be more conserved among viral species, which may facilitate the discovery of new inhibitors. On the downside, Nis require *in vivo* activation, such as phosphorylation, and must compete with high *in vivo* substrate concentrations. However, since Nnis target less conserved areas of the protein, they are more prone to the development of resistance. Some Nnis that can interfere with the functionality of the RdRp protein are reported below. Aurintricarboxylic acid (ATA, Figure 17B) is a compound capable of interfering with SARS-CoV-1 replication with an IC_50_ = 0.2 mg/mL by direct interaction with the RdRp. ATA was previously known for its ability to bind to several proteins including gp120 of HIV-1 and HIV-2 due to its anionic polymer characteristics. When applied to SARS-infected cell cultures, it inhibits mRNA transcription by more than 1000-fold with respect to control. The mechanism of action is associated with the inhibition of RdRp by the interaction of ATA.^227^ Other examples of non-nucleoside analogue inhibitors of RdRp protein have been reported by Dutta et al., who repurposed beclabuvir (Figure 17B) as a SARS-CoV-2 RdRp inhibitor.^228^ Beclabuvir is an inhibitor of HCV NSB polymerase^229,230^ that was only approved in Japan for the treatment of HCV infection as part of a triple combination therapy designated as Xymency. The authors of this study predicted a *K*_i_ of ∼50 nM for the binding of the compound to the protein active site.^228^ This result was achieved through docking studies against the homology model of RdRp from SARS-CoV-2, though experimental evidence of its effectiveness against SARS-CoV-2 still needs to be provided. Traditional Chinese medicine may represent a source of active compounds in the development and optimization of new active molecules to treat SARS-CoV-1 and -2. Examples of exploitation of natural products have been reported by Lung et al., who selected 83 molecules from traditional Chinese medicinal compounds exhibiting antiviral activity against SARS-CoV-1 and the corresponding compounds showing similar chemical structures as the results of a molecular docking study targeting RdRp of SARS-CoV-2, SARS-CoV-1, and MERS-CoV.^231^ Among these, theaflavin (ZINC3978446) (Figure 17B) can bind to the catalytic pocket near the active site of RdRp in SARS-CoV-2, SARS-CoV-1, and MERS-CoV. Theaflavin represents a starting point for an optimization of medicinal chemistry program and the mechanism of action.

### Perspective and Foreseen Studies: Alliance between Drug Discovery and Genetics

Genetics studies will improve our understanding of the mechanisms of action of viral and host proteins and will suggest the design of new drugs avoiding mutation-induced drug resistance or targeting failure. While genetics is widely used for the study of interspecies passage in coronaviruses, it is less employed for the discovery of new therapeutic targets,^232,233^ except for the invariant zone exploitation as proposed by Slanina et al.^234^ and which are at risk of the emergence of resistant variants. Several initiatives relied on other genetic concepts more specifically based on synthetic lethality properties. SLs represent pairs of gene or residues for which either mutation is neutral, but when the double mutation occurs, the protein is inactivated and the virus should be made unviable. Widely used to define cancer targets,^235^ the aim is to find a gene Y that is in a lethal synthetic relationship with gene X, whose mutation is responsible for the disease. This gene Y is a very good target because when it is inactivated by the attachment of a properly designed drug molecule, in a cancer cell with a mutated gene X, the cell dies. Inversely, its inactivation in a noncancer cell has no impact on this cell, making it possible to target only cancer cells.

How could we then extend these different concepts to the fight against coronaviruses?

**Figure 18:**
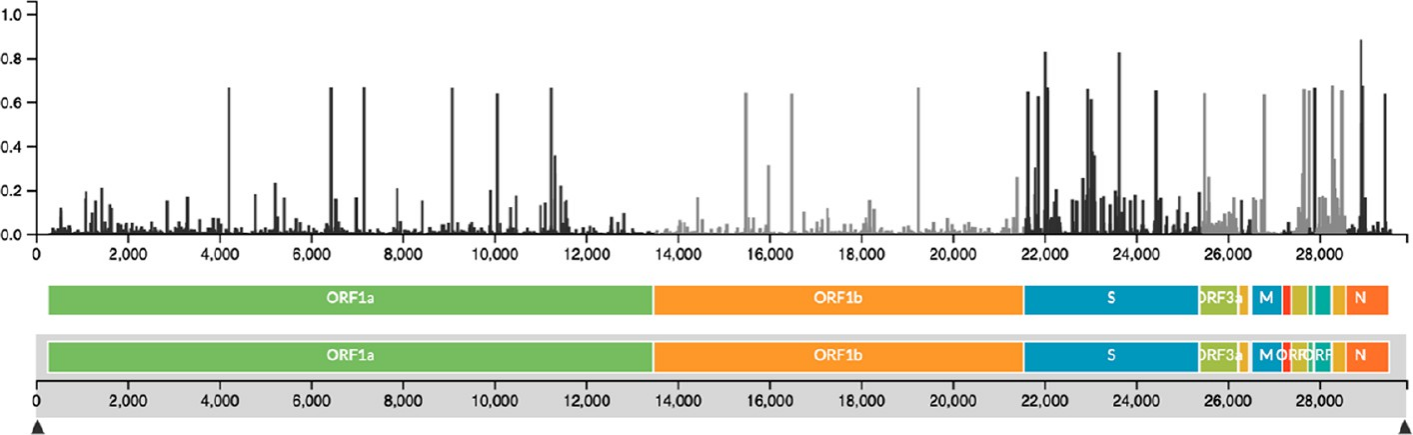
Mutational diversity. Number of aa mutations per position. Showing 3045 of 3045 SARS-CoV-2 genomes sampled worldwide (between December 2019 and December 2021). Diversity is shown by the Entropy level reported on the Y axis (range 0.0−1.0), codon number in protein gene of reference is indicated with the black spike characterized by a position (number on the x-axis) and entropy level (Y axis). Below in the horizontal line, the different gene considered important in the genomic diversity description are reported. The open reading frame 1a (ORF1a) gene in green was developed to specifically detect viral genomic RNA. The ORF1 is considered a marker of infectivity of the clinical specimens. Other indicated protein genes are S = spike protein gene, M = main protease gene. Adapted from https://nextstrain.org/groups/neherlab/ncov/global?c=clade_membership&p=grid&r=division.^237^

Understanding the mutational occurrence requires knowledge about the function of one core protein such as 3′–5′ exoribonuclease (nsp14-ExoN) allowing corrections of RNA replication errors, so the frequency of mutation appearances per nucleotide is reduced compared to other RNA viruses such as HIV or flu for example. But this frequency is increased by 3-fold in the coronaviruses compared to other viruses since their genome is on average 3 times larger than others (27 000–32 000 nucleotides) and constitutes the largest genome of RNA viruses. It is therefore necessary to solve a three-variable problem which is composed of the genome size, replication fidelity, and genome complexity. Indeed, RNA viruses must remain replicative with a high error rate, because while some viruses reduce their genome size (main RNA viruses), others such as coronaviruses reduce the appearance of mutations per nucleotide to reach a similar rate of mutation occurrence by genome.^236^Figure 18 shows the mutational diversity (mutational frequency at each amino acid position) of the SARS-CoV-2 genome as of December 10, 2021.

### Targeting Invariant Areas

The appearance of mutations thus seems to be an important advantage given to the virus, allowing it to resist a treatment or to improve its fitness.^238^ However, the vast majority of mutations are either neutral or deleterious:^239^ in the first case they do not provide better fitness but are not detrimental either, while in the latter the essential functions that make the virus nonreplicative are hampered.^240^ The positions at which mutations are so deleterious that the virus becomes nonreplicative are called invariant positions. There is therefore a low percentage of positive mutations. A balance has to be achieved between positive and deleterious mutations since large numbers of deleterious mutations would make the virus nonreplicative. The genetic study of these deleterious mutations makes it possible to define new therapeutic targets that will block an essential function, which will therefore be important to drastically reduce fitness, while avoiding viral escape.^238^ A bioinformatic study on the SARS-CoV-2 genome shows a particularly well conserved region, namely, KRSFIEDLLFNKV, that is located around one of the known cleavage sites of the SARS virus predictively required for virus activation for cell entry. This sequence could be used to make a synthetic peptide to constitute a future vaccine.^241^ Another region of the S protein in MERS-CoV or SARS-CoV is also highly conserved and therefore can constitute the sequence of a future synthetic peptide to produce a vaccine.^242−245^ Huber et al.^246^ were interested in the viral RNA rather than the proteins encoded by it, as a target for new therapeutic targets. The study identified a few conserved regions, one of which has partial homology with the avian flu virus.

The barriers to using invariant regions in defining therapeutic targets are of three types:

1.There are not enough invariant positions to define a therapeutic target, which must be close positions in space and on the surface of the protein.

2.The position seems invariant but the whole possible mutational space of the virus has not been explored, so it may still mutate, especially if a selection force is imposed.

3. It cannot be mutated alone, but the function can be recovered by mutating one or more other positions (case of compensatory mutations).

**Figure 19:**
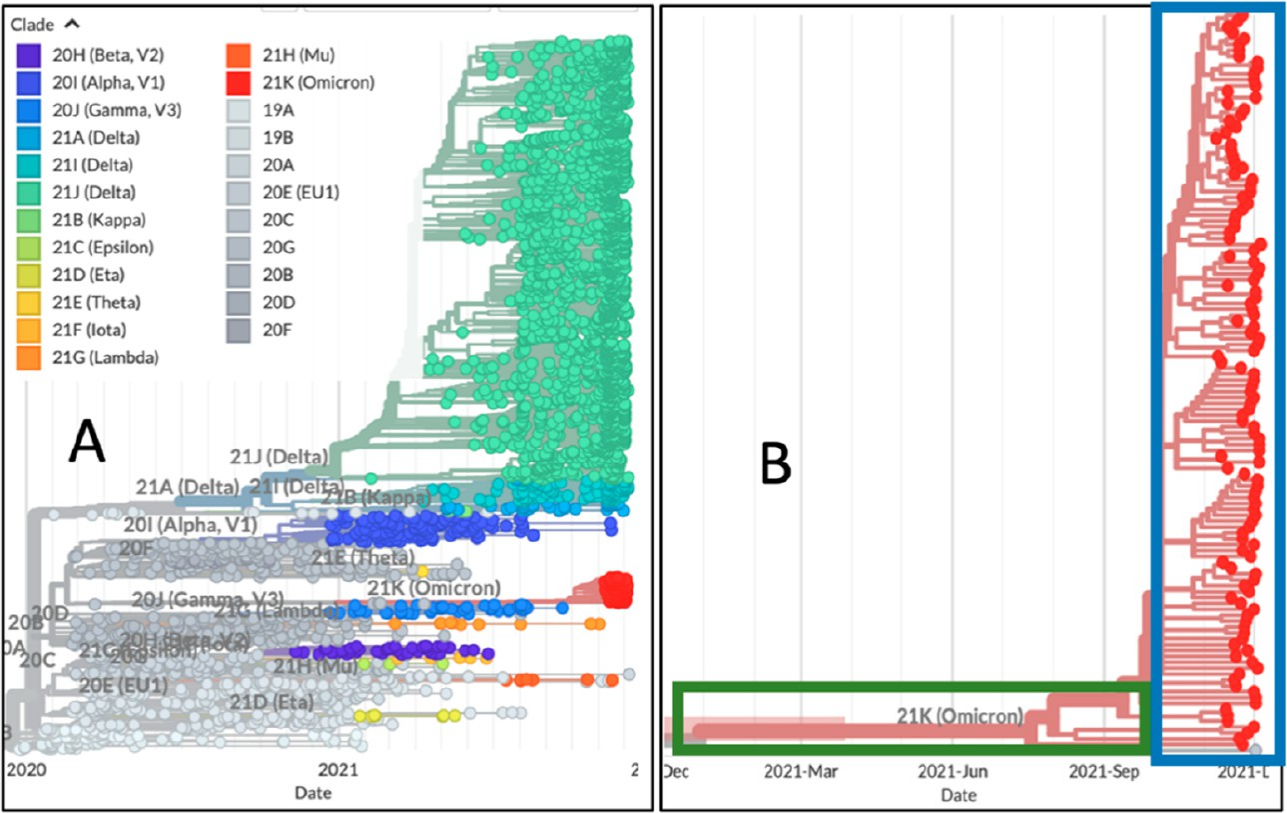
Snapshot of the phylogeny description of SARS-CoV-2 genomes sampled worldwide (between December 2019 and December 2021). In panel (A) this phylogeny shows evolutionary relationships of SARS-CoV-2 viruses from the ongoing novel coronavirus pandemic with a particular focus on Europe. Additional sequences from the rest of world were added. This picture shows how different outbreaks are connected and how the virus might have spread around the globe. Direct linkage on the tree does not necessarily mean there exists a direct epidemiological link. All data used (European and international) are deposited in GISAID. Each dot represents a variant case, isolated somewhere in the world. The different color indicates a different variants clade. The horizontal line indicates the divergence number. With time the divergence increases. (B) Phylogeny of SARS-CoV-2 genomes as in A, but Omicron variants only are shown. Adapted from https://nextstrain.org/groups/neherlab/ncov/global?c=clade_membership&p=grid&r=division.^237^

Figure 19 shows the accumulation of mutations which allows the construction of the phylogenetic tree of the different variants. Figure 19B shows the special case of the Omicron variant. Here we see that the Omicron variant started to emerge after accumulating mutations for 1 year (green box). Then we see that the population grows by enriching itself with new mutations that should improve its overall fitness (blue box). This accumulation of mutations can exemplify the second case described above.

### Intragenic Synthetic Lethality

As discussed above, the high mutability rate of RNA viruses makes it difficult to find conserved regions, whether they consist of residues distributed sequentially on the primary sequence (future synthetic peptide for a vaccine) or close in space to define a new possible future therapeutic target pocket. Previous studies on HIV used intragenic SL to enrich these areas of invariance and show that they could constitute new therapeutic targets avoiding escape.^247^ These zones can be defined from a sequence alignment of the protein to be targeted through a detailed study of the possible mutations (Vanet patent). After determination of invariant positions and pairs of coevolved residues, these can be classified either as the synthetic lethal (SL) or as the compensatory mutation (CM). Although these two kinds of position pairs appear as a result of coevolution, the causes of their appearance are quite opposite. In fact, a compensatory mutation allows the recovery of a function that disappeared due to a previous mutation. The emergence of compensatory mutations increases the variability of the genome. The cause of the appearance of SL pairs is of a completely different order: the two mutations that make up a pair of SLs are separately silent and only become lethal if they occur simultaneously. In genetic terms, they could be assimilated to an invariant pair, but mutating them individually does not result in a lethal phenotype. It is therefore the SL pairs we are interested in, if we wish to describe a target with increased global invariance. The invariant positions added of SL pairs constituting potential targets can be visualized on the protein 3D structure to identify those accessible on the surface. Finally, using pocket definition software packages, one can restrict the investigation to the solvent-exposed patches of residues most prone to bind small molecules.

This method was applied to different proteins of HIV-1, such as the protease, in which one of the two targets detected is lining a pocket necessary for the opening of the enzyme flaps. This mechanism is necessary for the peptide substrate to be correctly positioned in the active site. If the movement of these flaps is constrained by a small drug molecule, proteolysis is impaired, and the virus is inactivated.

The same approach applied to influenza allowed the discovery of three new targets.^248,249^ One of them is located in a loop involved in the spring-loaded mechanism. When the virus enters the endosome, the acidic pH induces a conformational change called the “spring mechanism” which rearranges the main loop into a linear α-helix allowing the fusion peptide to access the endosome membrane. As this mechanism initiates membrane fusion, allowing the integration of the viral genome into the host cell, blocking it with a small molecule, prevents viral infection. These newly defined targets may not be in the catalytic pocket of an enzyme, since allosteric sites often exert essential functions for viral replication. For example, the pocket described above, located under the flaps of the HIV protease, is not part of the major catalytic site that allows proteolysis but still blocks a function that is essential to the pathogenicity of the virus. Once these targets have been identified, the main task remains to design small molecules that could fit the pockets by molecular dynamics methods.^250^

### Extended Intragenic SL Strategy

Since synthetic lethal pair is defined by two positions whose simultaneous mutation only becomes lethal, intragenic SL strategy restricts these mutations to the same protein. Conversely, in the extended SL strategy, mutations are in two different proteins of the genome. This strategy can be used if two proteins carry a common function, as in the case of components of a protein complex, or if one of them is a substrate for the other. In either case, a drug located at the interface of both proteins could block their shared function.

In the case of the SARS-CoV-2 virus, M^pro^ is most closely linked to the other viral proteins whose maturation requires proteolysis. Thus, we could prevent an essential function, by blocking the link between M^pro^ and one of these viral protein substrates.^92^ By defining the SL pairs, one can design a small molecule targeting both proteins at the same time, so this extended strategy reduces the chances for the virus of escaping.

### Intergenic Synthetic Lethality

Viral proteins also have multiple links to infected host proteins,^13^ so we can use intergenic SL to block viral development. In fact, the virus can only replicate by infecting a cell by hijacking a number of cellular mechanisms that are essential for its function. The host genes encoding for the proteins involved in these processes and known as host-dependent factors are good therapeutic targets and have the main advantage of being insensitive to direct viral resistance mutations. This is the case of some proteins in the immune system,^251^ which cannot be targeted directly as their function is essential to the host and its loss would lead to strong side effects. Basler et al.^252^ propose to directly target the interfaces generated between these proteins and those of the virus to prevent viral replication. To avoid depriving the host of essential functions, one could target processes that are only triggered in the infected cells. In eukaryotic cells, many functions are redundant to avoid the catastrophic effect of a mutation affecting an essential function carried out by a single protein. These redundancies, when viral development depends on them, also keep the cell alive long enough to produce new virions. They can thus be targeted because two proteins share an SL relationship. In fact, if a virus blocks a host protein function, purposely designed drug molecules can simultaneously block the functionally redundant protein. This abolishes the function guaranteed by the two related host proteins only in infected cells, while the uninfected ones will still be able to use the redundant protein not targeted by the drug. Mast^92^ proposed several avenues using this strategy such as trying to block the virus-induced adaptive network states, the virus-mediated protein–protein interactions, or communal pathways. A general study on the possible host cell genes implicated in an SL intergenic relationship allowed discovery of 26 possible targeted genes,^253^ the most promising of which are the following: VKORC1, an anticoagulant that binds to ORF7a of SARS-CoV-2^254,255^ and targeted by warfarin;^256^ MED8, a gene required for the activation of transcription;^257^ translation initiation factor 4G (EIF4G), known for its role in cell growth, proliferation, and differentiation.^258^

Once one of these three genes is knocked out in the infected cells, the cellular multiplication rate decreases, whereas no change is observed in noninfected cells. Drugs targeting one of these proteins would hence prevent the viral development, since uninfected cells could still survive upon drug administration. Interestingly, EIF4G, was pinpointed by Mast^92^ as a possible target for another pathogen, the coxsackievirus, whose protease cleaves EIF4G and the poly-A binding protein.^259^

**Figure 20:**
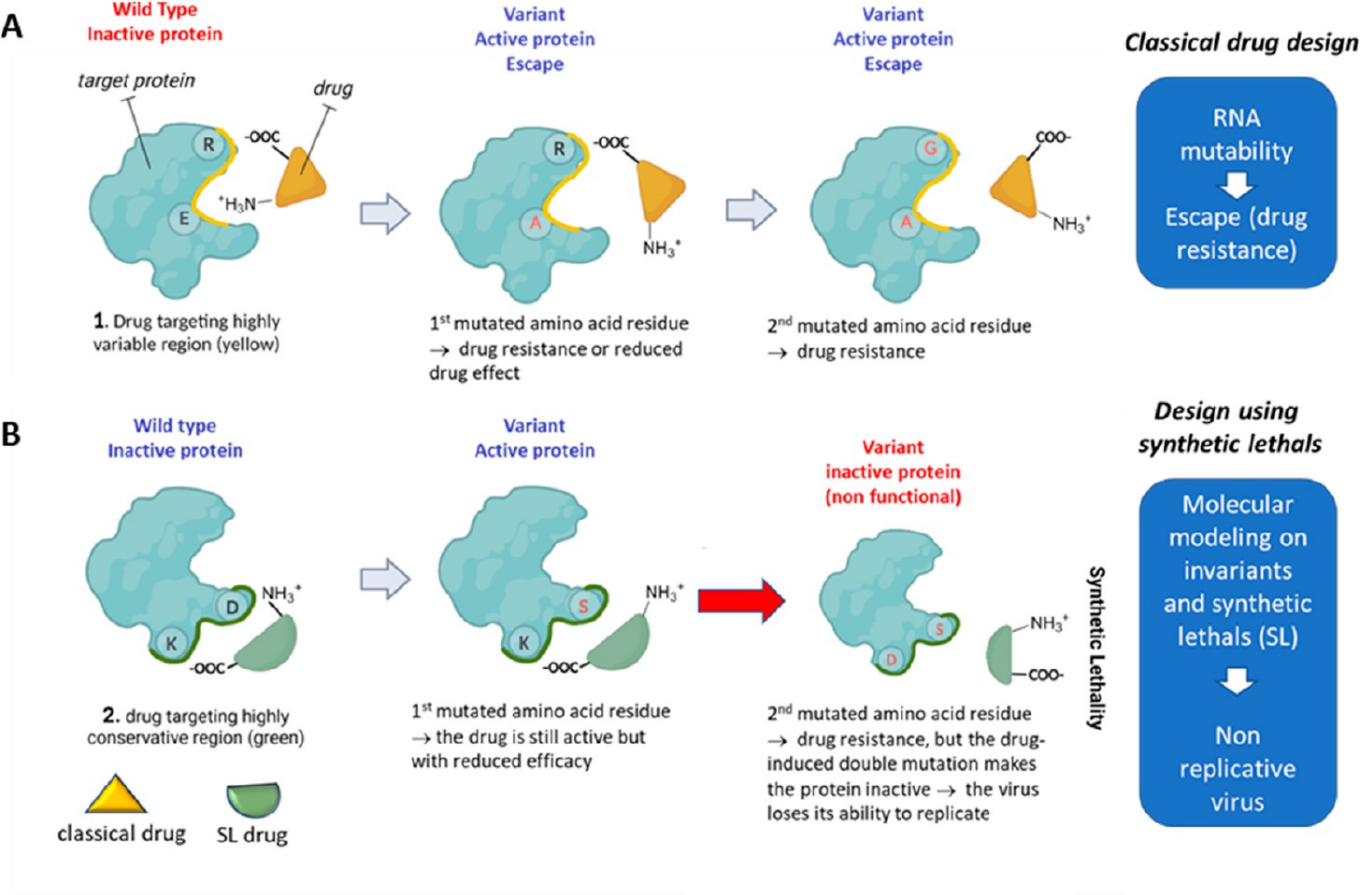
Schematic representation of intragenic SL strategy in comparison with classical drug design for antiviral drug development. (A) Classical drug design approach in which the drug binds to sites R and E typically in the active site in nonproperly selected zones. Progressively two mutations occur, E to A and R to G, causing drug resistance. (B) The molecules developed using the SL strategy, binding the invariant zones of virus wild-type proteins, may induce a double mutation (D to S and K to D), developing again a strategic drug resistance which leads to the inactivation of the affected protein and ultimately to the virus replicative failure.

The strength of the SL strategy is to identify invariant regions/residues of viral proteins that may constitute innovative target areas for the design of new long-lasting inhibitors against coronaviruses (and other RNA viruses) by limiting their potential for therapeutic escape (Figure 20). Accordingly, the proposed molecules have the potential to block viral replication, binding the invariant zones of the target viral proteins. In addition, they can counteract drug selective pressure by positively selecting mutants of the target residues insensitive to the treatment, but losing virus replicative capacity, ensuring the drug efficacy over time.

## Perspective

Although the development of vaccines and efforts paid for global vaccination campaigns have helped to contain SARS-CoV-2 spread and limited the impact risk factors to the development of severe COVID-19 disease, the progress in small-molecule drug discovery may also contribute to disease management. The initial identification of effective medications was mainly focused on repurposing agents with demonstrated activity against SARS-CoV-1, MERS-CoV, or correlated RNA viruses. Despite the intense efforts made to support this primary initiative, a very limited number of drugs were marketed.

Compounds targeting RdRp and M^pro^ enzymes, the most investigated targets since the first coronavirus outbreak in 2002, led the way in antiviral research by virtue of their specific domains and the lack of counterparts in human cells, which would ensure reduced risks of off-target adverse events. Against these targets remdesivir, molnupiravir, and nirmatrelvir–ritonavir have received Emergency Use Authorizations (EUAs), demonstrating efficacy and safety in the human challenge models.

As an antiviral drug may lose its effectiveness owing to virus resistance, improvement is still possible and the potential pan-coronavirus activity of these first-in-class therapeutics in cellular and animal models must be confirmed in humans.

The drug resistance phenomenon globally causes great concern: WHO and its international network of experts are monitoring changes to SARS-CoV-2 to understand how they may impact its properties, such as transmissibility and severity. The identification of amino acid changes in resistant mutants is an acknowledged stage in the mechanistic elucidation of any antiviral drugs. Despite the hundreds of mutations naturally occurring or induced by vaccines identified so far, no crucial key residues of antiviral drug targets seem to be involved, leaving SARS-CoV-2 mutants still sensitive to promising experimental or currently deployed therapies. Conceivably, the acute nature of SARS-CoV-2 infection requiring a short time course of antiviral therapy (such as for remdesivir compared to HCV and HIV infections) may undermine the role played by resistance to therapy; that remains a matter of some debate.

Understanding the mechanism behind the target structural basis (viral and host factors) and the related biological significance to virus replication is essential for the development of better targeted therapies, in the context of the ongoing epidemic of COVID-19 and/or future outbreaks of other coronaviruses.

A strong support to drug discovery may derive from genetics that improves our knowledge on the mechanisms of action of the viral and host proteins and provides a direction for the design of innovative drugs. These new compounds can avoid mutation-induced drug resistance or targeting failure, by preserving inhibitory activity against wild-type as well as evolutionary mutated strains. To this aim the search for intragenic synthetic lethality could be applied to SARS-CoV-2 proteins which accumulated mutations such as Rpdp, Rpdp accessory proteins, nsp5 main protease, and the S protein. All of them play crucial roles in the replication cycle of this virus, and intragenic synthetic lethality can be exploited against pockets carrying out essential functions.

To increase the impact on other circulating and possible future coronaviruses, a comprehensive work could take into account similar viruses such as SARS-CoV-1 and MERS-CoV. Several databases such as ViPR^260^ and GISAID^261^ store tens of thousands of coronavirus sequences which could be used for this work. Two different approaches should thus be considered: SL strategies can be applied separately to proteins of each single virus. Otherwise, sequences of all equivalent proteins from different viruses could be aligned together to identify multivirus SLs. Should the two strategies yield the same result, that will greatly reinforce the fact that the identified targets are important for all the viruses considered. Thus, a single drug molecule could be administered regardless of the epidemic, SARS-CoV-1, MERS-CoV, or SARS-CoV-2, and should be considered as the leading molecule if a new epidemic, arising from the emergence of a new coronavirus, was to occur.

In addition, novel strategies allowing work on the sequence/structure alignment of several proteins could be considered. Intergenic SLs have worked in the identification of new types of anticancer molecules. The same can be applied to the discovery of new antiviral therapies targeting pairs of proteins belonging to the host and the virus working in conjunction. Functional or structural links between proteins are indeed very good new therapeutic hot spots that can be identified by computers.

Innovative drug discovery programs should envisage antiviral screening plans including highly pathogenic coronaviruses (SARS-CoV-1, MERS-CoV, and SARS-CoV-2), endemic representative CoVs, and pre-emergent CoVs isolated from intermediate reservoir hosts (BatCoVs) that may continue to spill over into human populations, leaving global public health vulnerable to future sanitary emergencies. In a One Health perspective the genetic studies of multiple organisms should be applied and biologically validated to translate a genetic isolated concept in a genetic alliance with drug discovery approaches. Nonetheless, greater efforts should be devoted to building up adequate animal models (small animal models and nonhuman primate models) capable of recapitulating clinical signs in humans that will benefit the progress of a drug discovery pipeline toward the development of an effective and long-lasting therapy.

## Abbreviations Used

3CLpro: 3C-like protease
6-HB: six helix bundle
ADME: absorption, distribution, metabolism, and excretion
AIV: avian influenza A virus
ATA: aurintricarboxylic acid
CC_50_: 50% cytotoxic concentration
CD: circular dichroism
CHR: C-terminal heptad repeat
CM: compensatory mutation
CoV: coronavirus
CTSB: cathepsin B
CTSL: cathepsin L
dNHBE: differentiated normal human bronchial epithelial cells—EpiAirway
EC_50_: 50% effective concentration
EV: enterovirus
FP: fusion peptide
GBA: 4-guanidinobenzoic acid
GBPA: 4-(4-guanidinobenzoyloxy)phenylacetic acid
HAT: histone acetyl transferase
HDAC2: histone deacetylase 2
HR: heptad repeat
HTS: high-throughput screening
LLE: lipophilic ligand efficiency
MD: molecular dynamics
M^pro^: main protease
NHR: N-terminal heptad repeat
NiRAN: nidovirus RdRp-associated nucleotidyl transferase
Nis: nucleoside analogue inhibitors
NNis: non-nucleoside analogue inhibitors
nsp: nonstructural protein
NTP: nucleoside triphosphate
ORF: open reading frame
PEG: polyethylene glycol
P-gp: P-glycoprotein
PIKFYVE: FYVE finger-containing phosphoinositide kinase
PK: pharmacokinetics
PsV: pseudovirus
RBD: receptor binding domain
RdRp: RNA-dependent RNA polymerase
RDV: remdesivir
SL: synthetic lethal
SPR: surface plasmon resonance
TM: transmembrane
TMPRSS2: transmembrane serine protease, type 2
TRMT1: tRNA methyltransferase 1
USP: ubiquitin specific protease
VOC: variant of concern
